# Polyploidization and pseudogenization in allotetraploid frog *Xenopus laevis* promote the evolution of aquaporin family in higher vertebrates

**DOI:** 10.1186/s12864-020-06942-y

**Published:** 2020-07-29

**Authors:** Yanglei Jia, Xiao Liu

**Affiliations:** Fishery College of Zhejiang Ocean University, Key Laboratory of Marine Fishery Equipment and Technology of Zhejiang, Zhoushan, Zhejiang China

**Keywords:** Aquaporin, Amphibian, *Xenopus*, Polyploidization, Gene duplication, Pseudogenization, Evolution

## Abstract

**Background:**

Aquaporins (AQPs), as members of the major intrinsic protein (MIP) superfamily, facilitated the permeation of water and other solutes and are involved in multiple biological processes. AQP family exists in almost all living organisms and is highly diversified in vertebrates in both classification and function due to genome wide duplication. While some AQP orthologs have been lost in higher vertebrates through evolution.

**Result:**

Genome-wide comparative analyses of the AQP family between allotetraploid frog *Xenopus laevis* (*Xla*) and diploid frog *Xenopus tropicalis* (*Xtr*), based on the genome assemblies, revealed that the number of AQPs in *Xla* genome nearly doubled that in *Xtr* (32 vs. 19). Synteny analysis indicated that the distribution of the retained AQPs in *Xla* subgenomes (17 in *Xla. L*, the longer homeolog of *Xla* genome and 15 in *Xla. S*, the shorter homeolog of *Xla* genome) were highly symmetrical when compared with that in *Xtr* genome. Remarkably, two members in *Xla. L* and four members in *Xla. S* were lost through evolution. Blast analysis revealed that the lost AQPs in *Xla* are pseudogenized via either the deletion of some exons or some single nucleotide insertions or deletions that lead the reading frame shift. Additionally, comparative genomic analyses suggested that the orthologs of AQPs that with one copy absence in *Xla* are also prone to be lost in higher vertebrates.

**Conclusion:**

This study revealed that polyploidization and subsequent pseudogenization and deletion in *Xla* genome promote the evolution of AQP family in higher vertebrates. Besides, our results would also contribute to understanding the evolution of AQP family.

## Background

The channel proteins that selectively mediated the transmembrane transport of water and other small molecules through biological membranes are named as aquaporins (AQPs) [1, 2]. AQPs represent a superfamily that defined as major intrinsic proteins (MIP) due to that most of their functions are still unconfirmed. The molecular structures of AQPs share six transmembrane domains (TM-1 to − 6) that linked by five connecting loops (Loop-A to -E); importantly, they contains two highly conserved signatures [3]. The first is the highly conserved segments that named asparagine-proline-alanine (NPA) motif localized at Loop-B and -E [3, 4]. This signature is critical for the formation of hourglass structure of AQPs [3]. The other one is the aromatic/arginine region (Ar/R region) that composed of four amino acids residues (one in TM-2, one in TM-5 and two in Loop-E) [5, 6]. The aromatic amino acids in Ar/R region restrict the pore diameter of the channel and function as a selectivity filter. AQPs are present in almost all living organisms and play important roles in maintaining body osmotic balance and metabolism [7, 8].

Based on the structural signatures and functional diversity, AQPs are grossly divided into four subfamilies [9–12]. The first subfamily functions as a water-selective channel and is named as classical AQP (C-AQP) [9]. The second subfamily is glycerol permeable and is named as aquaglyceroporin (AQGP). The third subfamily refers to the members that have highly degenerative NPA motifs and the functions are still not yet been examined. This subfamily is named as superaquaporin or subcellular AQPs (SAQP) [10]. The last subfamily is a new one that is separated from the C-AQP subfamily and the members may mediate the transmembrane transport of ammonia and certain other molecules [12]. This subfamily is defined as AQP-8. The number of AQPs in different subfamilies is quite diversified among phylum [9]. Such strategic expression patterns and functional diversity of AQPs are the basis for adapting to environmental changes and evolution.

It is well known that whole-genome duplication (WGD) plays an important role in vertebrate evolution [13]. The common ancestor of early vertebrates before ray-fined fish have experienced two rounds of WGD [14–16]. Moreover, teleosts like zebrafish have undergone another round of WGD. Some fish families such as salmon even have totally experienced fourth rounds of WGD [17]. Consequently, these evolutionary events have also diversified the AQP family in fishes [18]. While the total number of AQP genes in mammals is relatively small when compared with that in fish families. These phenomena suggested some AQP orthologs have been lost in higher vertebrates during the process of evolution. So far, the pattern of the AQP family in vertebrates have only been performed in a few species, such as zebrafish and human.

Amphibians, as the origin of land vertebrates, are evolved from fish-like animals [19–21]. *Xenopus*, one of the most important families in amphibians, has aquatic larvae and breathes through gills like fish. Through a drastic process referred to as metamorphosis, the larvae transform into a terrestrial adult and breath through the lung during a relatively short period of time [22]. The metamorphosis process is also accompanied by a radical change of living environment. Importantly, AQPs play important roles for adapting to the environmental changes during the process of metamorphosis [12]. Until now, limited studies have been performed on the genome wide AQP genes in amphibia species. Recently, the genome of an allotetraploid frog *Xenopus laevis* (*Xla*) [23] and a diploid frog *Xenopus tropicalis* (*Xtr*) [24] that assembled at chromosome level have been published respectively. Evidence shows that the number of chromosomes in *Xla* (2 *N* = 36) nearly doubled that in *Xtr* (2 *N* = 20) and most other diploid frogs [23]. This data indicates that *Xla* has experienced an additional round of whole genome duplication [23]. As a tetraploid frog that arose via the diploid frog through genome duplication, both karyotypic and genomic sequencing data suggested that the genome of *Xla* could be divided into two distinct separated subgenomes or homeologs of different chromosomal size (shorter and longer) [23]. To make it convenient for the comparation analysis, an “L” and “S” was appended to represent the longer and shorter homeologs (*Xla.L* and *Xla.S*) in *Xla* genome respectively. Up to now, the distribution and correlation of AQPs in *Xla* and *Xtr* genome have never been studied. The good reference genomes for allotetraploid frog *Xla* and diploid frog *Xtr* provided the feasibility to analyze the evolutionary of the AQP families between them.

Here we de novo identified the complete AQP family in *Xla* and *Xtr* by utilizing the available genome sequence data. The phylogenetic analyses were performed to characterize the functionally critical signatures and to classify the AQPs in *Xenopus* species into distinct subfamilies. Moreover, the duplication and deletion of the AQPs between *Xla* and *Xtr* were further analyzed. Besides, the distribution and evolution of the AQP family in the other vertebrates were analyzed based on the comparative genomic study. Furthermore, the expression pattern of the AQP orthologs in various adult tissues and organs and at different developmental stages of *Xla* were examined by utilizing the RNAseq data. This study aims to provide comprehensive understanding of the AQP family in duplication and deletion during the process of evolution in vertebrates.

## Results

### Identification and phylogenetic analysis of the AQPs

A total of 32 AQPs were detected based in the whole *Xla* genome, with 17 members localized in *Xla. L* and 15 in *Xla.S*. As a comparison, a total of 19 AQP genes were detected in the *Xtr* genome, which was much less than the total number of AQPs in *Xla* genome (19 vs. 32) but more than the numbers that distributed in each of the separated homeologs in *Xla* genome (19 vs. 15 or 19 vs. 17). At the same time, the composition pattern and distribution of AQP orthologs in the other vertebrates were summarized (Additional file 1: Figure S1 and Additional file 12: Table S1). Remarkably, the total number of AQP genes in different amphibians were highly diversified when compared with the other vertebrates (Additional file 1: Figure S1 and Additional file 12: Table S1).

**Fig. 1:**
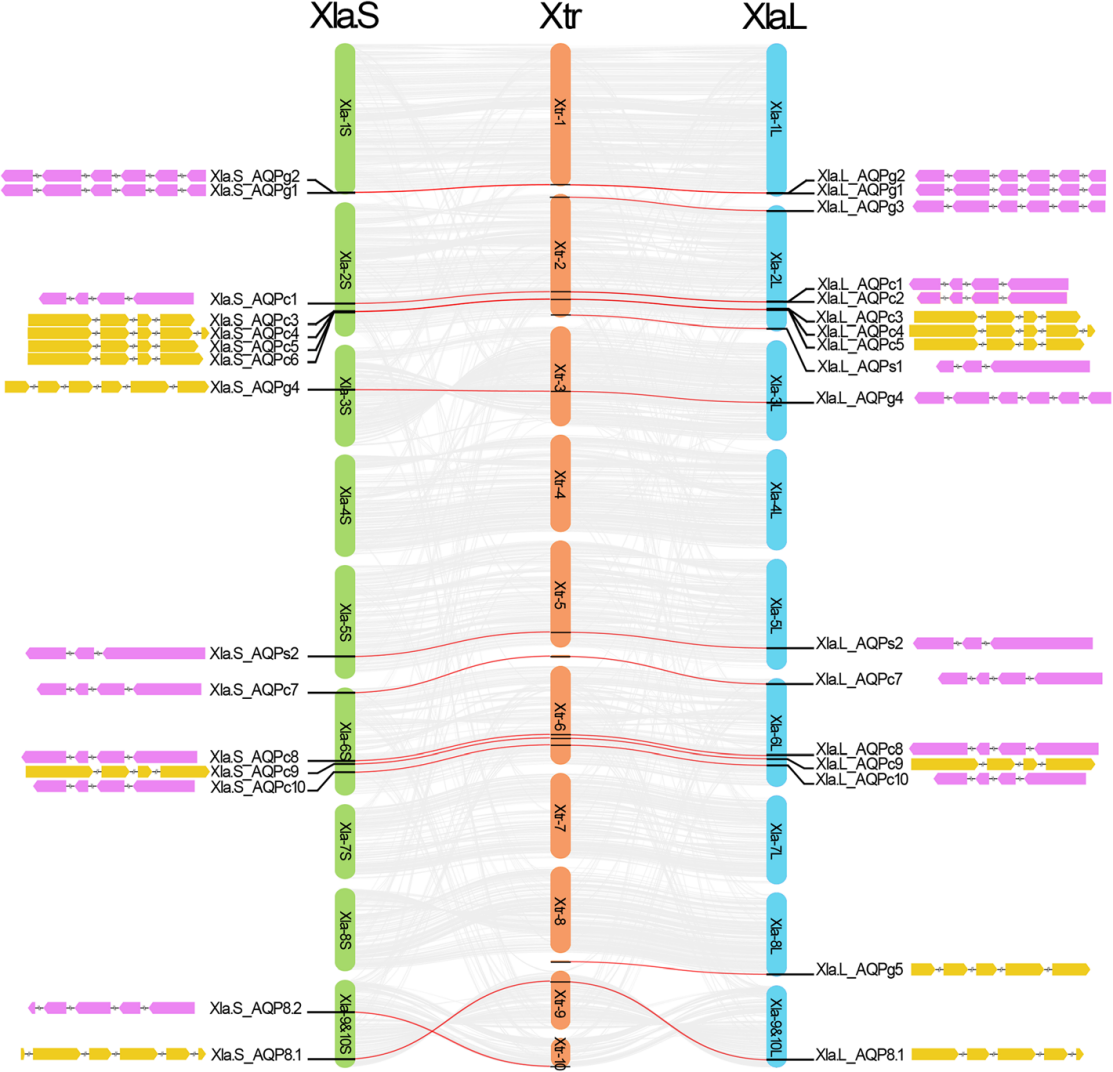
Synteny analysis between the homeologs in *X. laevis* and *X. tropicalis* genome. The linkages between the AQP genes were marked with red lines. Genome location and gene structure of the AQP genes in *X. laevis* genome were shown beside the chromosomes. The wedges colored in violet and yellow represent the exon regions in AQP genes. Different color represents the different encoding direction. The shrinked lines represent the intron regions

Chromosomal location analysis revealed that the 32 AQP genes in *Xla* genome were distributed across 14 of the 18 chromosomes (Fig. 1). As a comparison, the 19 AQPs in *Xtr* genome were distributed across 7 of the 10 chromosomes and two separated scaffolds due to the low-level assembly of the terminal regions (Fig. 1). As expected, the synteny analysis indicated that the AQPs in *Xla* genome were symmetrically distributed between *Xla. L* and *Xla. S*, which was consistent with the distribution of the AQPs in *Xtr* genome (Fig. 1). It should be noted that the distribution density of AQPs that localized on the second chromosome is the most (8 in *Xtr-2*, 7 in *Xla-2 L* and 5 in *Xla-2S*) in different homeologs when comparing that on the other chromosomes (Fig. 1).

**Fig. 2:**
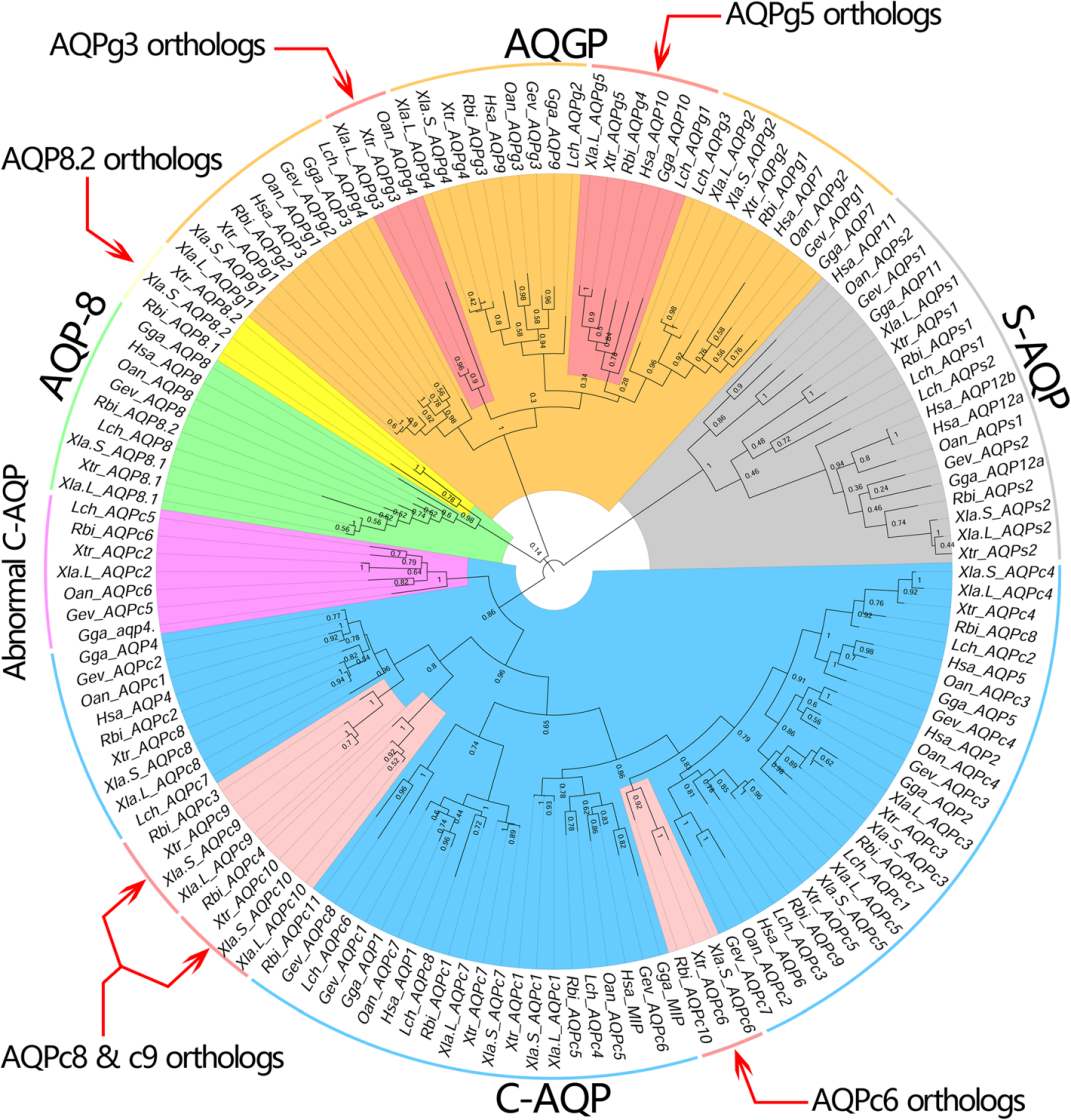
Maximum likelihood based phylogenetic tree of the AQP families in vertebrates. The AQP families were distinct separated into four clades (marked with different colors), representing the four subfamilies respectively. The protein sequences were collected from the following species: *Latimeria chalumnae* (*Lch*), *Rhinatrema bivittatum* (*Rbi*), *Xenopus laevis* (*Xla*), *Xenopus tropicalis* (*Xtr*), *Gopherus evgoodei* (*Gev*), *Gallus gallus* (*Gga*), *Ornithorhynchus anatinus* (*Oan*), *Homo sapiens* (*Hsa*)

The phylogenetic trees, constructed by both maximum likelihood (ML) (Fig. 2) and neighbor-joining (NJ) (Additional file 2: Figure S2a) methods using the AQP protein sequences, showed that the whole set of the AQPs were clearly clustered into four distinct subfamilies: C-AQP, AQGP, AQP-8 and S-AQP. As expected, the members localized at the consistent positions on the chromosomes in *Xenopus* species were clustered together. Since the AQP sequence is composed of two internal repeats, the phylogenetic relationship between the amino-terminal (N-ter) half and carboxy-terminal (C-ter) half of the protein sequences was also produced using the ML methods (Additional file 2: Figure S2b). Obviously, the N-ter and C-ter half of all AQP sequences were distinctly clustered into two separated semicircles. These data revealed that no internal swapping mutation between the N-ter and C-ter have been detected in all vertebrates AQP sequences. Multiple alignments revealed that the orthologs of AQP genes in different vertebrates were highly conserved, especially in the NPA motif domain and even in the S-AQP subfamily (Additional file 2: Figure S2c, d). Remarkably, except S-AQP subfamily, the other three subfamilies were all expanded in amphibians (Additional file 1: Figure S1 and Additional file 12: Table S1). To make the subsequent analysis more convenient, the AQP genes in *Xla* and *Xtr* were renamed depending on the chromosomal location and their subfamilies that they clustered. In addition, appending a “Xtr_” or “Xla.L_” or “Xla.S_” at the front of AQP gene names that identified from *Xtr* or the longer or shorter homeologs in *Xla* respectively.

### Duplication and deletion of the AQP genes in *Xenopus*

**Fig. 3:**
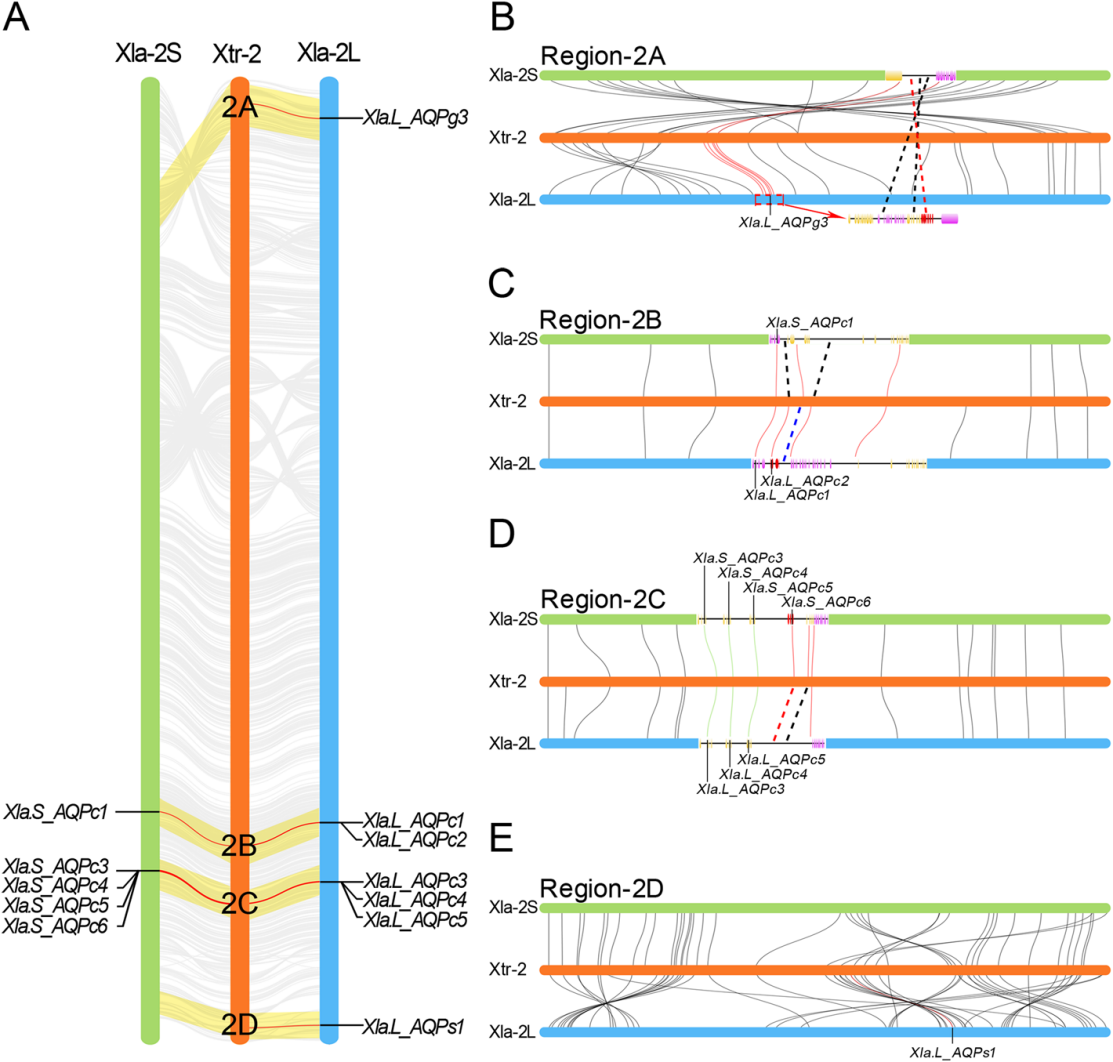
Deletion of AQP genes in the second pair chromosomes in *X. laevis*. **a** Synteny analysis of the second chromosomes in the homeologs in *X. laevis* and *X. tropicalis* genome. The regions encoding AQP genes were shadowed in light yellow. The linkages between AQP genes were marked with red line. AQP gene names in *Xla* genome were marked beside the second chromosomes. **b** Synteny analysis of region-2A encoding AQPg3 ortholog. **c** Synteny analysis of region-2B encoding AQPc1 and AQPc2 orthologs. **d** Synteny analysis of region-2C encoding AQPc3 to AQPc6 orthologs. **e** Synteny analysis of region-2D encoding AQPs1 ortholog. In this figure, red curves represent the linked genes were appeared in both *Xtr* and the corresponding homeologs in *Xla*. Red dashed lines represent the genes that appeared in *Xtr* genome but absented in *Xla* genome

It is obviously that two members in *Xla. L* homeolog (Xla.L_AQPc6 and Xla.L_AQP8.2) and four members in *Xla. S* homeolog (Xla.S_AQPg3, Xla.S_AQPc2, Xla.S_AQPs1 and Xla.S_AQPg5) were absent during the process of evolution when compared with the corresponding locations of the AQPs in *Xtr* genome (Fig. 1). Coincidentally, the vast majority of the absented AQP genes were also distributed on the second pair of chromosomes. Synteny analysis of the second chromosomes indicated that there are several inverted regions in both the longer and shorter homeologs of *Xla* when compared with the corresponding region in *Xtr* (Fig. 3a). Furthermore, some multiple inverted regions even occurred in *Xla-2 L* and *Xla-2S* when compared with the *Xtr-2* chromosome.

Elaborate synteny analyses were performed to investigate the deletion of AQPs in the second chromosome in *Xla. S* and *Xla. L* homeologs. Remarkably, the gene that encoding AQPg3 in *Xla-2S* was localized at an inverted region (Region 2A) when compared with *Xtr-2* (Fig. 3a, b). While in *Xla-2 L*, the corresponding region was highly collineated and localized at the initial terminal. Sequence blast indicated the sequence encoding AQPg3 was not lost completely in *Xla-2S*. The first exon of Xla.S_AQPg3 still exists with two gaps (Additional file 3: Figure S3a). Further analysis suggested that except the deletion of AQPg3 in *Xla-2S*, the sequences encoding the other two genes at the downstream of AQPg3 (Golgi pH regulator and GTP-binding protein 8) were also lost at the same time (Fig. 3b).

The genes encoding the orthologs of AQPc1 and AQPc2 were tandemly localized at the same region (Region 2B) in the second pair of chromosomes (Fig. 3a). Synteny analysis suggested this region was highly collineated in both the *Xla-2S* and *Xla-2 L* when compared with *Xtr-2* (Fig. 3c). Sequence blast indicated that the exons encoding AQPc2 were completely lost in *Xla-2S*. Further analysis suggested that combined with the deletion of AQPc2 in *Xla-2S*, another gene (Glutaminases: liver-type) next to AQPc2 was also lost at the same time. Interestingly, the gene encoding SPRYD4, which localized between AQPc2 and Glutaminases, was lost in *Xla-2 L* but appeared in *Xla-2S* (Fig. 3c). These data implied that the gene deletion in *Xla* genome was selective rather than random.

The genes encoding AQPc3 to -c6 were tandemly localized at the adjacent position of another region (Region 2C) in the second pair chromosomes (Fig. 3d). Arrangement of the genes in this region in *Xla-2S* and *Xla-2 L* was highly collineated when compared with *Xtr-2* based on the result of synteny analysis. Sequence blast suggested that the second and fourth exons of that encoding AQPc6 still existed in *Xla-2 L* (Additional file 3: Figure S3b). Surprisingly, an in-frame stop codon (TGA) was identified in the second exon. Moreover, one gap with seventeen nucleotide residues and two separated sites with single nucleotide residue insertion, which lead the shift of reading frame, were detected in the fourth exon of AQPc6 in *Xla-2 L* when comparing the consistent nucleotide and derived amino acid sequence in *Xla-2S* and *Xtr-2* (Additional file 3: Figure S3b). Except the deletion of AQPc6 in *Xla-2 L*, another uncharacterized neighboring gene was also lost at the same time.

The gene encoding AQPs1 was localized at the end terminal of the second chromosomes (Region 2D) (Fig. 3a). It should be noted that this region was multiple inverted in both *Xla-2S* and *Xla-2 L* when compared with the corresponding region in *Xtr-2* based on the result of synteny analysis, especially in the short homeolog (Fig. 3e). Additionally, the ortholog of AQPs1 was localized at the edge of the inverted segment. Sequence blast revealed that the nucleotide sequences which encoding AQPs1 in *Xla-2S* were completely lost during the process of evolution. Moreover, synteny analysis also suggested that some genes that appeared in *Xla-2* homeologs but lost in *Xtr-2* in this region. Further analysis revealed the sequences encoding the lost genes in this region in *Xtr-2* were localized in some scaffolds (Additional file 4: Figure S4a). These data suggested that the assembly quality at the end terminal region of the second chromosome in the *Xtr* genome was relatively low.

**Fig. 4:**
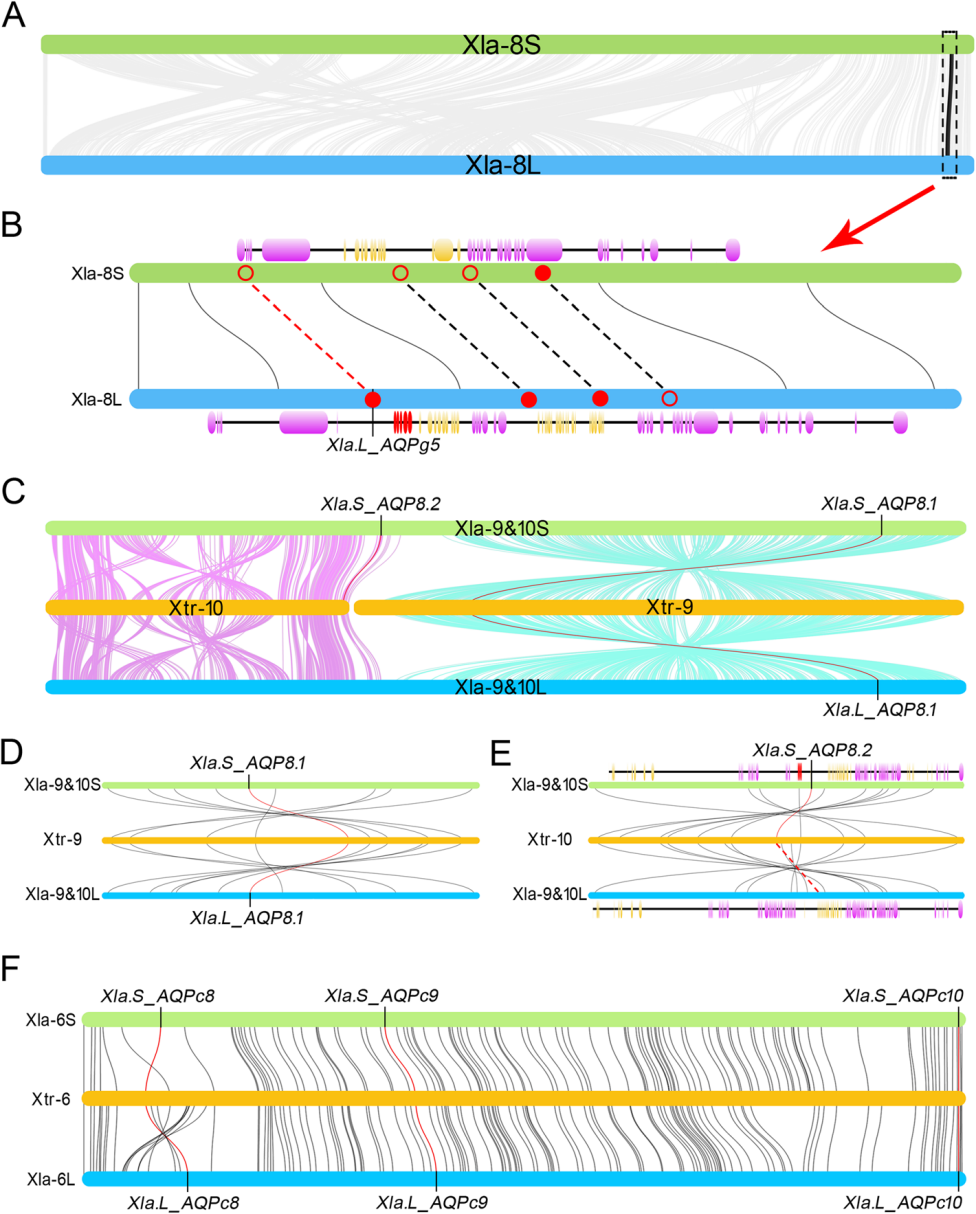
Synteny analysis of the AQP orthologs in *Xenopus* species. **a** Synteny analysis of the eighth pair chromosomes in the homeologs in *X. laevis*. **b** Synteny analysis of the region encoding AQPg5 ortholog. **c** Synteny analysis of the ninth pair chromosomes in the homeologs in *X. laevis* and the ninth and tenth chromosomes in *X. tropicalis* genome. **d** Synteny analysis of the region encoding AQP8.1 ortholog. **e** Synteny analysis of the region encoding AQP8.2 ortholog. **f** Synteny analysis of the region encoding AQPc8 to AQPc10 orthologs. In this figure, red curves represent the linked genes were appeared in both *Xtr* and the corresponding homeologs in *Xla*. Red dashed lines represent the genes that appeared in *Xtr* genome but absented in *Xla* genome

Like the location of AQPs1 in the second chromosome, the gene encoding the orthologs of AQPg5 were also localized at the end terminal of the eighth chromosome. The assembly quality of the end terminal in *Xtr-8* was also relatively low (Additional file 4: Figure S4b). Some scaffolds were not attached to the chromosome sequence. As a result, the sequence encoding Xtr_AQPg5 was localized in a separated scaffold (NW_016683840.1) (Fig. 1 and Additional file 4: Figure S4b). Therefore, synteny analysis was only conducted between *Xla-8 L* and *Xla-8S*. It is notably that the corresponding regions that encoding AQPg5 were highly collineated in eighth pair chromosomes in the *Xla* genome (Fig. 4a). Further analysis suggested that except the deletion of AQPg5, another two genes (SH2 domain-containing adapter protein E (SHE) and tudor domain-containing protein 10 (TDRD10)) that localized at the downstream of AQPg5 were also disappeared at the same time in *Xla-8S* (Fig. 4b). Additionally, the gene encoding neuronal acetylcholine receptor beta-2, localized next to TDRD10 in *Xla-8S* was also absent in *Xla-8 L*. Sequence blast indicated that the nucleotide sequence encoding AQPg5 in *Xla-8S* was partially lost (Additional file 3: Figure S3c). Except the initial three exons that were lost during the process of evolution, the other three were completely retained. Whereas, in-frame stop codons (TAA) were detected in the fifth and sixth exons respectively (Additional file 3: Figure S3c). Additionally, a deletion of thymine residue, which leads to reading frame shift, was also detected in the sixth exon (Additional file 3: Figure S3c).

Interestingly, the ninth pair of chromosomes in *Xla* genome were originated from a fusion of proto-chromosomes of *Xtr-9* and *Xtr-10* (Fig. 4c) [23]. The front one third part (Part-A) of the ninth pair chromosomes in *Xla* homeologs was corresponding to *Xtr-10* (Fig. 4c). Remarkably, synteny analysis revealed that dozens of inverted regions were detected in Part-A of the ninth pair chromosomes. Some regions contain complex inversions when compared with the corresponding region in *Xtr*. In contrast, the other two thirds parts (Part-B) was corresponding to *Xtr-9* (Fig. 4c). Unlike the front part in genes arrangement, these parts were completely inverted in both the *Xla. L* and *Xla. S* when compared with the corresponding region in *Xtr* (Fig. 4c). Interestingly, the gene arrangements in the ninth pair of chromosomes in *Xla* genome were extensively collineated when compared with each other according to the results of synteny analysis (Additional file 4: Figure S4c).

The genes encoding AQP8.1 and AQP8.2 in *Xtr* genome was localized in the ninth and tenth chromosomes respectively (Fig. 4c). Consequently, the AQP8 orthologs that localized at Part-A in the ninth pair of chromosomes in *Xla* genome were named AQP8.2. In contrast, the other orthologs that localized at Part-B were named as AQP8.1 (Fig. 4c). Additionally, no collinearity was detected between the separated regions encoding the orthologs of AQP8.1 and AQP8.2 independently. These data implied that the two orthologs in the AQP8 subfamily were evolved independently and not derived from duplication. As expected, both AQP8.1 and AQP8.2 were localized at the inverted regions (Fig. 4d, e). Sequence blast implied that the exons which encoding AQP8.2 were retained in *Xla-9&10 L* (Additional file 3: Figure S3d). While a gap that contained six nucleotide residues but led no reading frame shift was detected in the first exon. Meanwhile, a long block deletion was detected in the third exon. Additionally, stop-codons were also detected in the gap area in different reading frames (Additional file 3: Figure S3d). Moreover, two additional in-frame stop codons were detected at the downstream of the gap in the third exon. As an exception, the other two exons were completely reserved (Additional file 3: Figure S3d).

Except the regions that mentioned above, the members that retained completely in *Xla* genome were also analyzed. Unlike the distribution of AQPc3 to -c6 that localized at the adjacent position in the second chromosomes (Fig. 3d), AQPc8 to -c10 were tandemly localized in the sixth chromosomes but were separated by a string of genes respectively (Fig. 1 and 4f). Synteny analysis suggested that the regions encoding AQPc8 to -c10 orthologs were extensive collineated in *Xla* homeologs in comparison to the corresponding region in *Xtr-6*, although a small-scale region encoding AQPc8 were inverted (Fig. 4f). Additionally, these data also showed that the string of genes separating AQPc8 and -c9 were not highly collineated with the genes that localized between AQPc9 and -c10 (Fig. 4f). Therefore, these data implied that the abundant AQPs in the sixth chromosomes were not derived from tandem duplication.

### Evolution analysis of the AQPs in vertebrates

Comparative genomic studies between different vertebrates were conducted to explore the evolution of AQPs. It is obvious that the genome sequences of different vertebrates were highly collineated (Additional file 1: Figure S1). The regions encoding AQP genes were extensively conserved when compared with each other, especially for the tandemly duplicated orthologs.

**Fig. 5:**
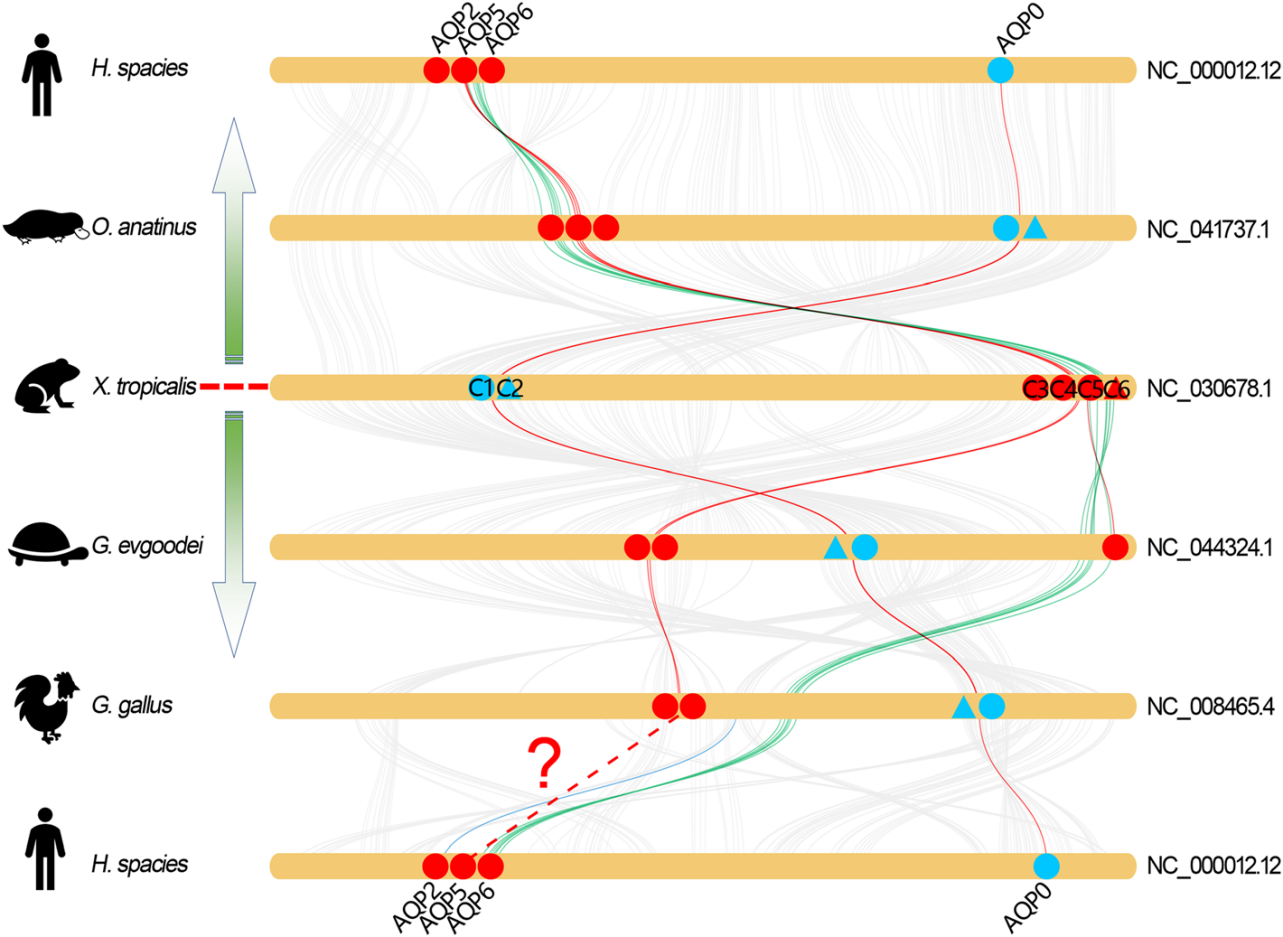
Collinearity relationship between the orthologs from AQPc1 to -c6 in different species. The round-corner rectangles filled in yellow represent the conserved regions of the chromosomes in different species. The words behind the round-corner rectangles represent the number of chromosomes. The circles filled in blue represent the conserved Mip protein. The triangles filled in blue represent the abnormal C-AQPs that neighbor localized beside Mip. The circles filled in red represent the orthologs of AQPc3 to -c5. The triangle filled in red represent the orthologs of AQPc6. The red curves represent the linkages between AQP orthologs. The green curves represent the linkages of the inverted region

Remarkably, the C-AQP subfamily is distinctly expanded in amphibian species (Additional file 1: Figure S1 and Additional file 12: Table S1). The regions encoding the orthologs of AQPc1 to -c2 and -c3 to -c6 were localized at two separated regions in the same chromosome and were highly conserved across different vertebrates (Fig. 5). As an exception, the number of AQP genes in these two regions were reduced in higher vertebrates. Synteny analysis revealed that the ortholog of AQPc2 was absent in human genome but was retained in the other species (Fig. 5). In contrast, the orthologs of AQPc1 were retained in all organisms. Similarly, the ortholog of AQPc6 were also lost in higher vertebrates. Interestingly, synteny analysis suggested the evolution of the region encoding AQPc3 to -c6 diverged after amphibians. This region is inverted in *G. evgoodei* genome, unlike the corresponding region in *Xtr* genome. Coincidentally, the orthologs of AQPc3 to -c6 were localized at the edge of the inverted region. As a consequence, the four tandemly duplicated AQP genes in *Xtr* were separated into two groups in the *G. evgoodei* genome (Fig. 5). Additionally, synteny analysis between *G. evgoodei* and *G. gallus* indicated that the arrangement of the genes in this region were highly conserved. While in mammals, this region was highly conserved except the deletion of AQPc6 orthologs (Fig. 5).

**Fig. 6:**
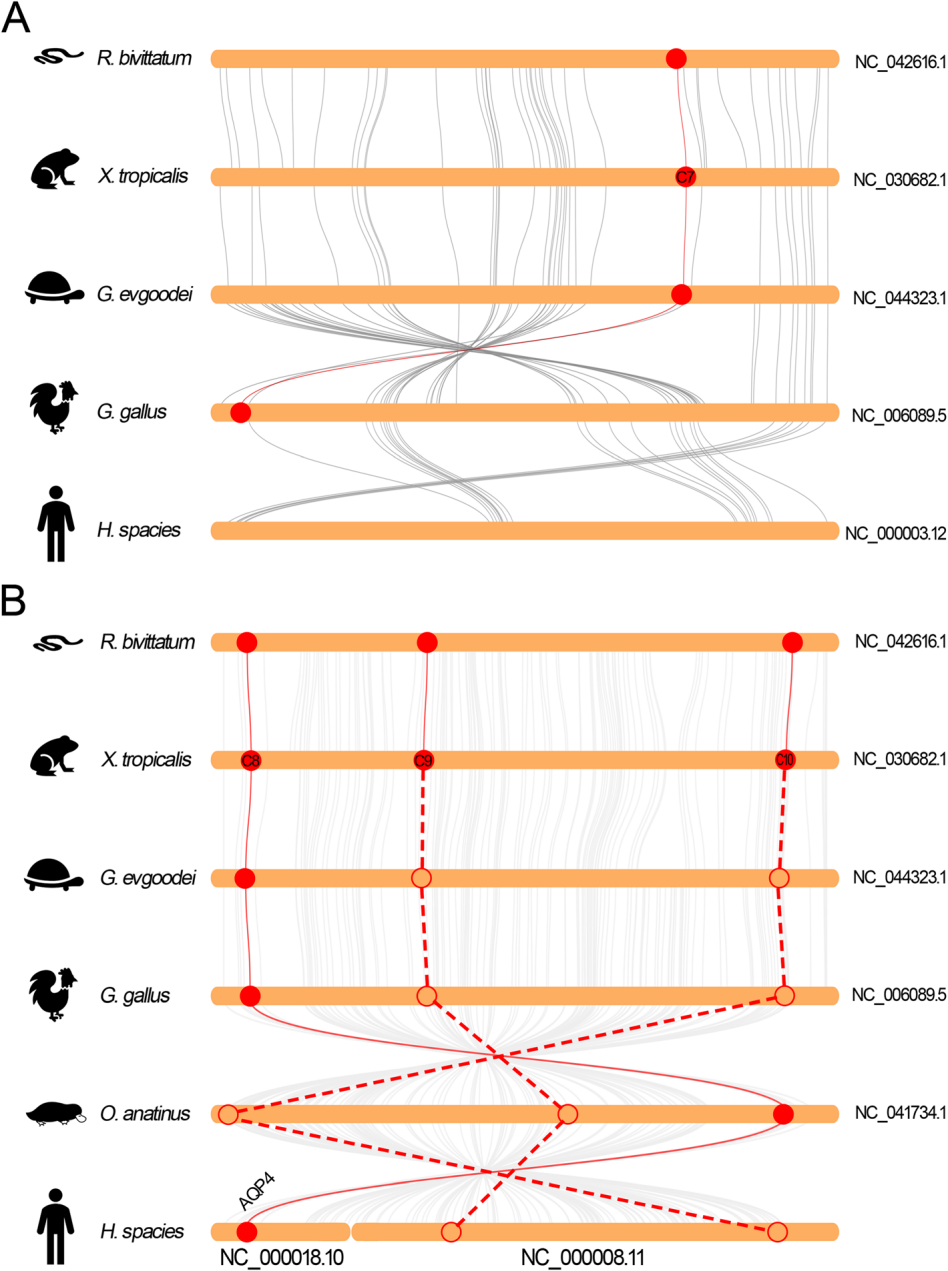
Collinearity relationship between the orthologs of AQPc7 (**a**) and AQPc8 to -c10 (**b**) in different species. The round-corner rectangles filled in yellow represent the conserved regions of the chromosomes in different species. The words behind the round-corner rectangles represent the number of chromosomes. The circles filled in red represent the AQP orthologs appeared in the corresponding chromosome. The hollow circles represent the lost AQP orthologs in the corresponding chromosome. The red curves represent the linkages between the AQP orthologs. The dashed lines represent the imaginary linkages between the lost genes

As shown above, the orthologs of AQPc7 and AQPc8 to -c10 were localized at the same chromosome and were separated by a long block of protein coding genes in *Xenopus* species (Fig. 1). Similar arrangement of these orthologs were also occurred in the members of Sauropsida (Fig. 6). Moreover, the corresponding regions in these species were highly conserved. In contrast, extensive chromosomal rearrangements and inversions were occurred in mammals during the process of evolution (Fig. 6a). Unexpected, the ortholog of AQPc7 in human genome was disappeared in this conserved region and was appeared in another chromosome that showed low collinearity with the other vertebrates (data not show).

As mentioned above, the region that encoding AQPc8 to -c10 were tandemly localized and separated by several genes in *Xenopus* species (Fig. 4f). Comparative genomic studies showed that this region in different vertebrates were highly conserved (Fig. 6b). As an exception, the corresponding region in human genome was separated into different chromosomes. Interestingly, the orthologs of AQPc8 were presented in all species. However, the orthologs of AQPc9 and -c10 only appeared in amphibians. Remarkably, this region showed extensive collinearity in the genome of amphibians and reptiles as well as birds, with no inverted region was detected between them (Fig. 6b). In addition, the genes that neighbor localized beside the orthologs of AQPc9 and AQPc10 were retained and no long block deletion of protein coding genes were detected in higher vertebrates. These data revealed that the orthologs of AQPc9 and -c10 were simply deleted in higher vertebrates during the process of evolution. Likewise, the arrangement of the C-AQPs like the orthologs of AQPc8 to -c10 in *Xenopus* was also not detected in fish families like zebrafish and even in *Latimeria chalumnae*, a species that belonging to lobe-finned fish and more closely related to tetrapods than to ray-finned fish.

**Fig. 7:**
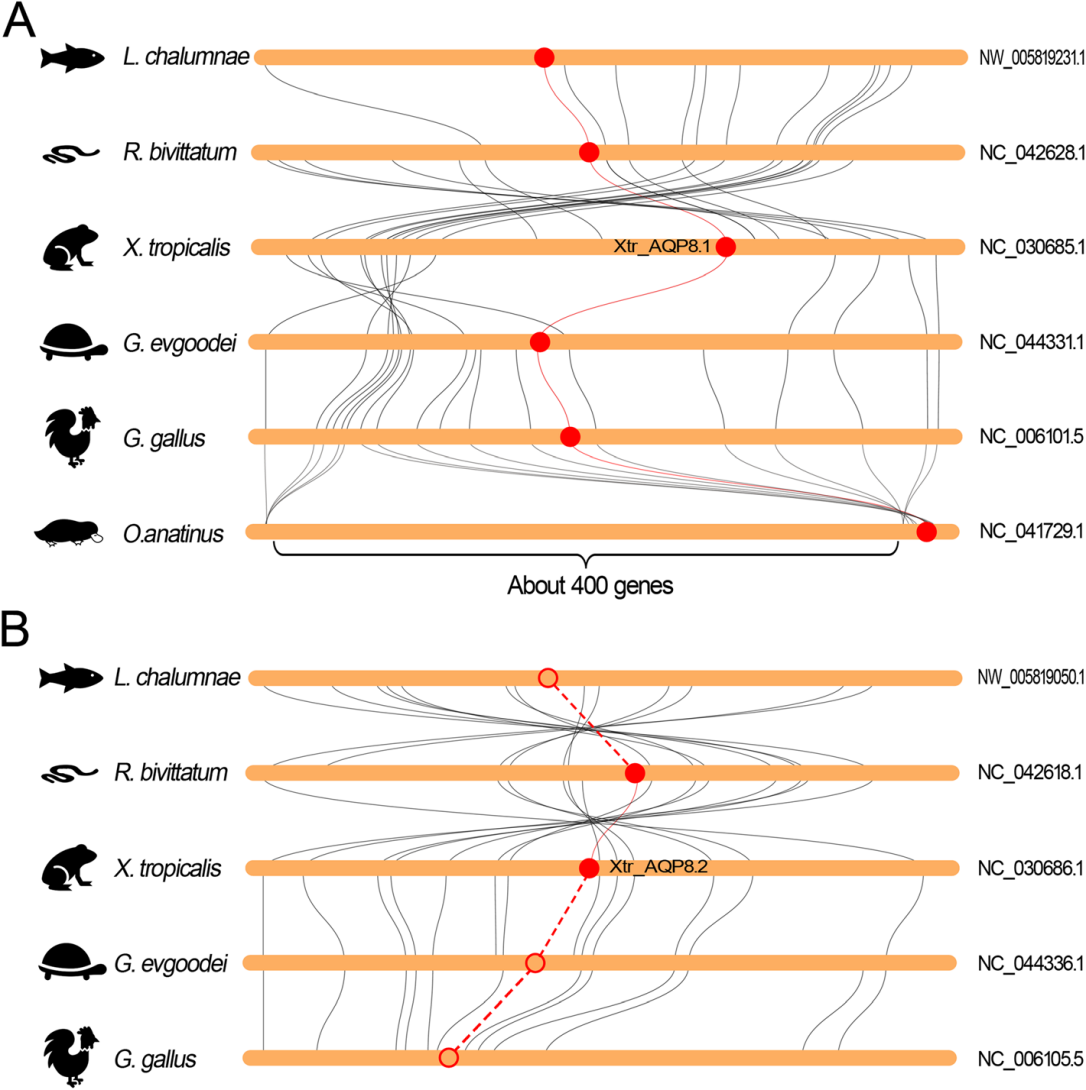
Collinearity relationship between the orthologs in AQP-8 subfamily in different species. **a** Collinearity analysis of AQP8.1 orthologs. **b** Collinearity analysis of AQP8.2 orthologs. The round-corner rectangles filled in yellow represent the conserved regions of the chromosomes in different species. The words behind the round-corner rectangles represent the number of chromosomes. The circles filled in red represent the AQP orthologs appeared in the corresponding chromosome. The hollow circles represent the lost AQP orthologs in the corresponding chromosome. The red curves represent the linkages between the AQP orthologs. The dashed lines represent the imaginary linkages between the lost genes

Remarkably, the AQP-8 subfamily in amphibians were also expanded in comparison to other vertebrates (Additional file 1: Figure S1, Fig. 2 and Additional file 12: Table S1). The orthologs of AQP8.1 and AQP8.2 were localized at the separated chromosomes in the *Xtr* genome (Fig. 1, Fig. 4 and Fig. 7). As a contrast, these two orthologs were localized at the separated regions of the same chromosome due to the fusion of the ninth and tenth chromosomes (Fig. 4c). Comparative genomic studies showed that these two regions were highly conserved in higher vertebrates and even in *L. chalumnae* (Fig. 7). As in *Xtr* genome, these two regions in other species were also localized at the different chromosomes or scaffolds. Synteny analysis implied that the orthologs of AQP8.1 appeared in all species including *L. chalumnae* (Fig. 7a). In contrast, the orthologs of AQP8.2 absented in the conserved regions in all species except in amphibians (Fig. 7b).

**Fig. 8:**
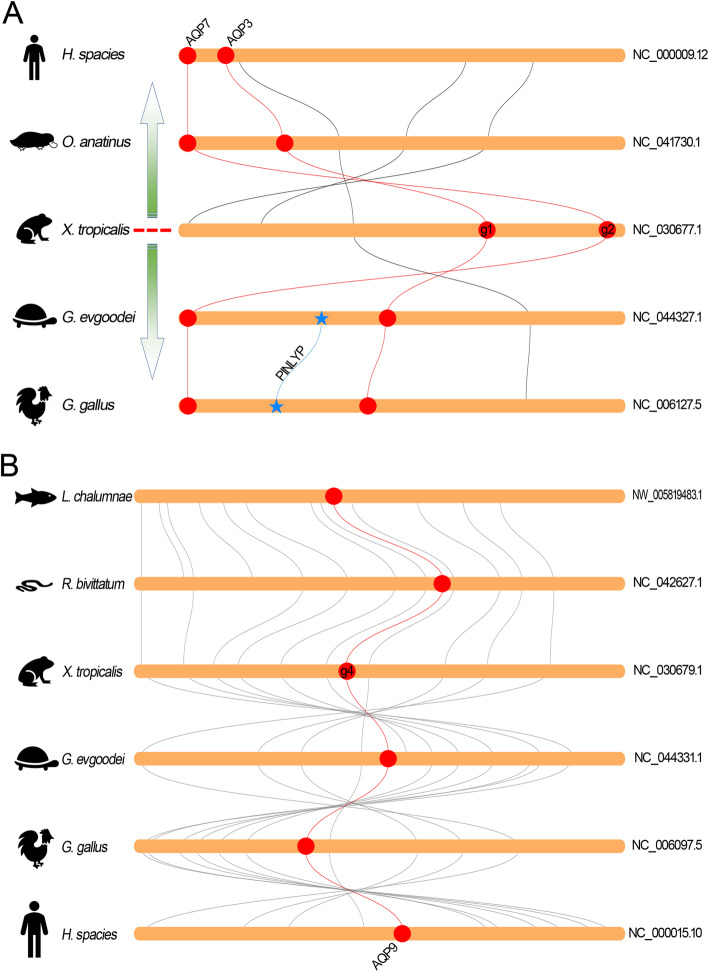
Collinearity relationship between the orthologs in AQGP subfamily in different species. **a** Collinearity analysis of AQPg1 and -g2 orthologs. **b** Collinearity analysis of AQPg4 orthologs. The round-corner rectangles filled in yellow represent the conserved regions of the chromosomes in different species. The words behind the round-corner rectangles represent the number of chromosomes. The circles filled in red represent the AQP orthologs appeared in the corresponding chromosome. The red curves represent the linkages between the AQP orthologs. The blue stars represent the inserted gene between the orthologs of AQPg1 and -g2. The blue curve represents the linkage between the inserted gene

The members in AQGP subfamily in *Xenopus* species was also expanded when compared with the higher vertebrates. Comparative genomic studies showed that the orthologs of AQPg1 and AQPg2 in vertebrates were colocalized and were highly conserved (Fig. 8a). Interestingly, in Sauropsida species like reptiles and birds, these two orthologs were separated by a protein encoding gene (phospholipase A2 inhibitor and LY6/PLAUR domain containing protein, PINLYP). Besides these two orthologs in AQGP subfamily, the region encoding the orthologs of AQPg4 were also show extensive collinearity in vertebrates (Fig. 8b). In contrast, no collinearity has been detected between the remaining two orthologs in vertebrates (data not show).

**Fig. 9:**
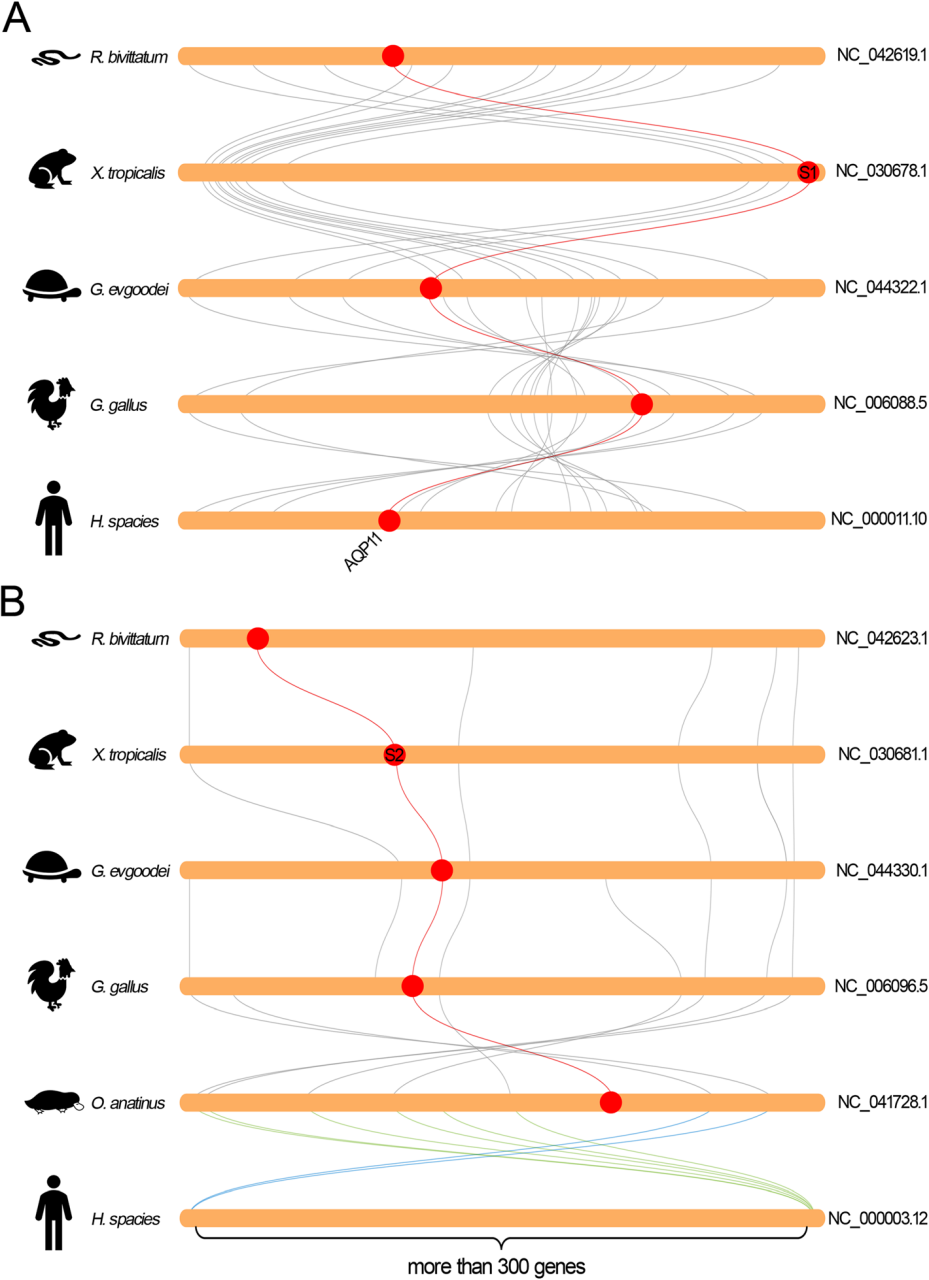
Collinearity relationship between the orthologs in S-AQP subfamily in different species. **a** Collinearity analysis of AQPs1 orthologs. **b** Collinearity analysis of AQPs2 orthologs. The round-corner rectangles filled in yellow represent the conserved regions of the chromosomes in different species. The words behind the round-corner rectangles represent the number of chromosomes. The circles filled in red represent the AQP orthologs appeared in the corresponding chromosome. The red curves represent the linkages between the AQP orthologs

Similar analyses were also produced on the members in S-AQP subfamily, the one that was not expanded in *Xenopus* species in comparison with the higher vertebrates. It is obvious that these two orthologs in vertebrates were highly conserved based on the synteny analysis results (Fig. 9a, b). As an exception, the conserved region encoding AQPs2 in human genome were localized at two separated regions due to inter-chromosomal rearrangements and inversions (Fig. 9b). Additionally, the ortholog of AQPs2 in human genome was disappeared in this conserved region and has been translocated to another chromosome (Additional file 1: Figure S1). Moreover, the rearrangements of this region in human genome are speculated to cause the tandem duplication of AQP12 (AQP12a and AQP12b).

### Structure and pole analysis of the AQPs in *Xenopus*

Clustal analyses were conducted on the complete CDS and amino acid sequences of the AQP genes in *Xla* and *Xtr* genomes. Obviously, the identities among both CDS and amino acid sequences in the complete set of AQP genes were quite low (Additional file 5: Figure S5 and Additional file 6: Figure S6). While the identities between the orthologs were extensively high (more than 90%). In C-AQP subfamily, the identity of the orthologs that localized at the adjacent positions in chromosome were higher (more than 50%) than those localized separately; this is true even for the orthologs of AQPc8 to -c10 that were separated by several genes respectively (Fig. 4f). As an exception, the identity between AQPc1 and -c2, though localized next to each other is relatively low (about 30%). In contrast, the AQP genes that clustered into different subfamilies could be clearly distinguished by the sequence identities (less than 40% in CDS and less than 30% in amino acid sequence, Additional file 5: Figure S5 and Additional file 6: Figure S6). Nevertheless, the identity between orthologs of AQPs1 and AQPs2 that clustered into the S-AQP subfamily were relatively low (about 30%). These data imply the low overall amino acid homology of the members in the S-AQP subfamily in *Xenopus* species.

Multiple alignments of both CDS and amino acid sequence of the AQP genes indicated that all members contain two highly conserved regions corresponding to the two NPA motifs at the re-entrant helixes in Loop-B and -E (Additional file 7: Figure S7 and Additional file 8: Figure S8). The patterns of amino acids composition in NPA motifs were summarized based on the alignment of amino acid sequences (Additional file 13: Table S2). It should be noted that the complete set of the AQP orthologs contain an extensively conserved NPA motif except AQPs2. In addition, almost all members of the AQP family in *Xla* and *Xtr* genomes contain two conserved NPA motifs (Asn-Pro-Ala) except for AQPg2 (NSA-NPA), AQPg5 (NPA-NPT), AQPs1 (NPS-NPA) and AQPs2 (NSA-NPT or NSA-NPM). Moreover, the pattern of the second NPA motif in the ortholog of AQPs2 was diversified in *Xla* genome (Additional file 13: Table S2). It should be noted that the AQP members in different subfamilies could not be clearly distinguished by the pattern of the NPA motifs.

As the key filter of AQP channel, the amino acids composition of the Ar/R region in AQP protein sequence is critical for the specificity of permeation. Therefore, the amino acids composition of the Ar/R region in different members were summarized at the same time (Additional file 13: Table S2). As expected, the amino acid sequences of the Ar/R region among the orthologs are extensively conserved. Importantly, the amino acid sequences in Ar/R region, in contrast to the NPA motifs, are very useful for clustering the members into different subfamilies. In addition, the last amino acid is highly conserved in all subfamilies and appeared as Arg except the ortholog of AQPs1.

The composition patterns of Ar/R region in the members that clustered into C-AQP subfamily were typically appeared as “FHAR” or “FHCR” except AQPc2, which is appeared as “AAGR”. It should be noted that the first two sites were highly conserved (Phe and His) in C-AQPs. The common signature of these two amino acid residues was the large side-chain (benzene ring in Phe and imidazole in His), which extend to the inner surface of the channel in AQP and restrict the diameter of the pore. Additionally, the hydrophilic properties of His lead the members in this subfamily more suitable for the permeation of water molecules. Unexpectedly, both the conserved Phe and His at the first two sites were all replaced by Ala in AQPc2. Pore pattern analysis indicated that these amino acid replacements in the Ar/R region of AQPc2 could enlarge the channel diameter at the filter region (Additional file 9: Figure S9a-c).

As a subfamily that evolved to facilitate the specific permeation of glycerol, the members in AQGP subfamily were typically containing a channel wider than the C-AQPs. Consequently, the composition pattern of the amino acids in the Ar/R region of the members that clustered into AQGP subfamily typically appeared as “FGYR” or “FGCR” or “GGYR”. It is obvious that at least one Gly, the simplest α amino acid, appears at the first two sites of the Ar/R region in this subfamily. In addition, the hydrophilic amino acid (His) is missing. Pore pattern analysis indicated that the channel of AQPg5, whose Ar/R region contains two Gly residues at the first two sites, were larger than those that contain only one Gly (Additional file 9: Figure S9d, e).

In the AQP-8 subfamily, recently separated from the C-AQP subfamily, the first two amino acids of Ar/R region are typically appeared His and Ile. Importantly, the conserved His that localized at the H_5_ site in C-AQP subfamily switched to the H_2_ site in AQP-8 subfamily. In addition, the members with different patterns of amino acids composition of the Ar/R region showed entirely different channel diameters at the corresponding area (Additional file 9: Figure S9f, g). It should be noted that the diameter of AQP8.2 at the Ar/R region was significantly enlarged when compared with AQP8.1. While the rest of the region is highly conserved. The difference in pore diameter between AQP8.1 and AQP8.2 implied their functional differentiation during the process of evolution.

Due to the lack of structure template for the members clustered into S-AQP subfamily, the pore patterns of AQPs1 and -s2 orthologs were not analyzed in this study. Moreover, considering the highly diversification of the S-AQPs in both the sequence and structure, swiss-model is not a good way to analysis the pore pattern of them.

### Gene expression analysis of the AQPs in *Xenopus*

The expression patterns of the AQPs were evaluated using the RNAseq data from 23 oocyte/developmental stages and 14 adult tissues and organs; the heatmaps were created to display the expression pattern of the AQP members at different developmental stages (Additional file 10: Figure S10) and in different adult tissues (Additional file 11: Figure S11), with expression levels represented by transcripts per million (TPM) values. Surprisingly, the expression level between the ortholog genes were extensively asymmetric both at different embryo development stages and in different adult tissues. While no obvious preference was observed in selective expression between the two homeologs in *Xla*.

As we know that the stage of ovulation and fertilization and entire development of embryo must be maintained under water. It is obvious that the expression patterns of AQP members throughout the embryo development stages could be clearly separated into two sections at the stages of gastrula except Xla.L_AQPs1 that was maintained at a high expression level throughout the development stages. It should be noted that the expression pattern of AQP genes in the development stages from oocyte to blastula were extremely similar to that in the tissue of ovary except the expression level of the orthologs of AQPc7 were sharply declined from the unfertilized egg. It is notable that Xla.L_AQPg3 was maintained at an extensive expression level during this period. Except that, the expression level of the AQPg4 orthologs were gradually increased with the maturation of oocytes. While in unfertilized eggs, the expression level of the pair of AQPg4 were sharply declined. Unexpectedly, after fertilization that combining with sperm, the expression pattern of Xla.L_AQPg4 was recovered while the ortholog copy (Xla.S_AQPg4) was kept expressed at a low level. Interestingly, the members that clustered into C-AQP and AQP-8 subfamilies were not expressed or expressed at a quite low level after fertilization during this period. The extensive expressed AQGPs during this period may play important roles for the absorption of nutrition for embryos. Combined with the expression of Xla.L_AQPs1, only a few members of AQP are expressed in these stages. Briefly, the expression level of these AQPs in embryos change little until to the stage of gastrula. This data suggested that the expression of AQPs in the initial stages of embryonic development were inherited from oocytes and named as maternal genes.

When the embryo developed into the gastrula stages that the cells begin to differentiate, the expression level of the members that maintained in the blastula stages were sharply declined except Xla.L_AQPs1. This stage is relatively short in duration throughout the embryonic development (NF-10 to NF-12). Then the embryo developed into the neurula stages (NF-14 to NF-20). The expression pattern of the members that declined in gastrula stages were still maintained at low level. Interestingly, several new members that clustered into the C-AQP subfamily (orthologs of AQPc10 and AQPg1) begin to express at a high level gradually during these stages. With further development, the embryo changed from neurula to tailbud, a stage that characterized by the appearance of blood islands and olfactory placodes [25]. During these stages, the expression of the pair of AQPg4, which had sharply declined in the neurula stages, were gradually recovered to the original level successively. Additionally, the orthologs of AQPc1 and AQPc7 begin to express at a high level at the end of these stages. These expression patterns were maintained until the last stages of embryo development that heartbeat started and the tadpole is ready to hatch [25]. As an exception, the expression level of Xla.L_AQPg3 was suddenly increased at the end of embryo development.

During a developmental process called metamorphosis, the aquatic larval tadpole transforms into a partly terrestrial frog [26]. Not only the living environment, but also the breathing patterns and nutrient metabolism were all undergone dramatic changes. Depending on the expression pattern of AQP family members in different tissues, it can be concluded that some members that were not expressed or expressed at trace amounts throughout the stages of embryonic development, were abundantly expressed in some tissues followed by the differentiation. Just like the members that clustered into AQP-8 subfamily were not expressed throughout the development stages. While in adult tissues, the orthologs of AQP8.1 were widely expressed in several tissues. However, the expression patterns of this pair genes were disparate. Especially in the intestine and skin, the orthologs of AQP8.1 were abundantly expressed respectively. This data implied that the function of these ortholog genes were highly diversified.

As a member that expressed throughout the embryo development stages, Xla.L_AQPs1 was also expressed across the adult tissues. This data suggested that Xla.L_AQPs1 was an essential member for different cells to maintain the normal functions. In this condition, the gene could be named as “house-keeping AQP”. Interestingly, the ortholog pairs of AQPc7 that maintained at an abundant expressed level from the end of tailbud stages were also expressed across the adult tissues. Although a subtle distinction existed between the expression level of them. These data suggested that the ortholog of AQPc7 should also be regarded as “house-keeping AQP”. While the orthologs of AQPc1, which first expressed simultaneously with the pair AQPc7 at the stage of tailbud, were only abundantly expressed in eyes. Although one of them that localized in the longer homeologs was also expressed at a low level in other tissues. These data indicated that the orthologs of AQPc1 were also specifically expressed in tailbud stages and differentiation of visual organs exactly started at these stages.

It should be noted that the members that clustered into AQGP subfamily were highly expressed in the organs that functions as nutrient digestion, absorption, and reabsorption like stomach, intestine and kidney respectively. In contrast, the members that clustered into the C-AQP subfamily were poorly expressed in these organs except the house-keeping orthologs of AQPc7 that were highly expressed in most tissues. While in skin, the first barrier for amphibians to against the external invasions on land, the majority of the highly expressed AQPs were clustered into the C-AQP subfamily. These members may play important roles for skin to maintain moisture. Same expression pattern also occurred in the lung, the organ that functions as gas exchange. In addition, the orthologs of AQPg4 and AQPs2 were abundant expressed in liver and pancreas respectively. These expression patterns suggested these members may play important roles in metabolism. Interestingly, the expression of AQPc2, which contains the untypical pattern of the Ar/R region, was not detected, or expressed at a trace level throughout the development stage and different adult tissues. Briefly, the expression patterns of the complete set of AQP family showed obvious functional diversification in different tissues or organs, and jointly maintained the homeostasis of the organism.

## Discussion

In vertebrates, the AQP family was highly diversified due to whole genome duplication [9, 27]. While the total number of AQP genes in mammals is relatively small when compared with that in fish families. Some AQP genes were lost in higher vertebrates during the process of evolution. Since gene duplication promote the evolution of genome [13], the analyses of AQP genes between the genome of a tetraploid frog *Xla* and diploid frogs *Xtr* and the other higher vertebrates provide a novel insight into the evolution of AQP family.

### Duplication and deletion of AQP genes in *Xenopus*

*Xla* is a tetraploid frog that is proposed to be originated from interspecific hybridization of ancient diploid frogs. The synteny analysis revealed that fusion of chromosome-9 and -10, large inversions and extensive intra-chromosomal rearrangements have occurred in both the ninth pair homeolog chromosomes in *Xla* genome when compared with the homeolog chromosomes in *Xtr* genome respectively (Fig. 4c). Remarkably, previous research indicated that the fusion of the ninth pair of chromosomes in *Xla* occurred prior to allotetraploidization [23]. Additionally, the extensive collinearity between *Xla. L* and *Xtr* genome indicated that the separated subgenomes in *Xla* were arise from two distinct diploid progenitors [23]. While synteny analysis between the ninth pair of homeolog chromosomes in *Xla* genome showed extensive collinearity throughout the chromosomes (Additional file 4: Figure S4c). These data suggested the distinct diploid progenitors in *Xla* genome may evolved from a common ancestor that are later than *Xtr* and contains nine pairs of chromosomes (2 *N* = 18). It is obvious that the common ancestor was evolved from *Xtr* via the fusion of the ninth and tenth chromosomes. The low-quality assemble of the fusion region in the ninth and tenth chromosomes (Additional file 4: Figure S4d) in *Xtr* prevent us to explore the detailed mechanism between them.

As for two distinct diploids progenitors in *Xla* genome, the longer homeolog has more collinearity with the *Xtr* genome. About 40% of the protein-coding genes that duplicated by allotetraploidization have been lost asymmetrically in the *Xla* genome [23]. Additionally, more extensive gene deletion has been detected in the *Xla. S* homeolog (31.5% vs. 8.3%). The distribution of AQP genes in the separated homeologs (15 in *Xla. S* and 17 in *Xla.L*) was consistent with the above conclusion when compared with the number (19 in *Xtr*, 21.1% vs. 10.5%) in *Xtr* genome. However, the retention rates of the complete AQP family (32 of 36, about 84.2%) was much higher than the average rate in the whole genome. Importantly, the lost AQP genes in *Xla* were not belong to the same orthologs. These data suggested that at least one copy of the AQP orthologs in *Xtr* genome have been retained in *Xla* genome. The composition pattern of AQP family in the *Xla* genome ensures the maintenance of normal physiological metabolism. Synteny analysis suggested that the deletion of AQP genes in *Xla* genome were always accompanied with several adjacent genes. These phenomenon in the AQP family was not consistent with most of the deleted genes in *Xla* genome [23]. Considering the limitation and drawbacks of sequencing technology that some mistakes or even omissions in genome sequences will be possible. Moreover, considering the lack of the knowledge on the function of the AQP genes and the corresponding deleted neighboring genes, the correlation between them have never been reported and worthy of further research.

Blast analysis implied that most deletions of the AQP genes in *Xla* genome were not complete. Some exons of these absented members were still retained in *Xla* genome. Except simple deletion, most of them were pseudogenized by single nucleotide insertion or deletion and that leads to shift of reading frame or in-frame stop codon (Additional file 3: Figure S3). Remarkably, the pseudogenization processes of the AQP genes in *Xla* genome are gradual loss of exons as units. These data implied that the pseudogenes in *Xla* represent a transitional stage via deletion. Pseudogenization represents a rapid way for AQP family in *Xla* to revert to singly copy after polyploidization. Besides pseudogenization of the ortholog genes, selection of expression dosage is another efficient way to rapidly revert to single copy [28]. It is obvious that the expression level between the orthologs of AQPs in *Xla* genome were asymmetric both in different tissues and throughout the embryonic development stages. Considering the identity between the ortholog AQPs were higher than 90% (Additional file 5: Figure S5 and Additional file 6: Figure S6). Moreover, the composition pattern of amino acids in the signature regions (both in the NPA motifs and Ar/R region) were extensively consistent between the ortholog AQPs in *Xla* genome (Additional file 13: Table S2). These data suggested the low possibility of neofunctionalization or subfunctionalization between the AQPs orthologs. Mutation of the regulatory elements may be a reason for the reduced expression level of the ortholog copy of AQPs [23].

### Evolution of the AQPs in higher vertebrates

Interestingly, the pattern of the Ar/R region in the orthologs of AQPc2 that clustered into C-AQP subfamily showed atypical amino acid composition (AAGR) in *Xenopus* species. In general, the AQP genes that possess this composition pattern of Ar/R region are prevalent in the plant genome and clustered into the NIP subfamily, which is specialized from C-AQPs [29]. Remarkably, except the C-AQP subfamily, the other three subfamilies were all absent in plants [30]. As a compensation, the function of some members that clustered into C-AQP subfamily were converted and make plant C-AQPs functionally comparable to animals AQP family [31, 32]. Moreover, the presence of NIP subfamily in plants may have come from bacteria by horizontal gene transfer [33]. It is notable that the first two amino acids, conserved in the Ar/R region, are replaced by Ala in AQPc2, which enlarged the diameter of the channel at the filter region (Additional file 9: Figure S9a-c). In addition, the replacement of the conserved hydrophilic His may cause these orthologs to lose the capability to facilitate the permeation of water molecules [6]. Therefore, the orthologs of AQPc2 allow the permeation of molecules other than water. While in the genomes of *Xenopus* species, the four subfamilies of AQPs all appeared. These data indicated that the presence of the AQPc2 orthologs were not functional as a compensation for the absence of the other subfamilies in *Xenopus* species.

Besides plants, the orthologs of AQPc2 also exists in all vertebrate lineages except hagfishes and eutherian mammals [12, 34]. Like the member (XP_005174182.1) that clustered into the C-AQP subfamily and showed the similar Ar/R region (AIAR) in zebrafish, a species that ancient than amphibians. Additionally, blast analysis also suggested the gene encoding Mip (AQP0, lens fiber major intrinsic protein) protein was adjacently localized to it. However, in eutherian mammals, the abnormal C-AQPs was not exist (Fig. 5). In contrast, the neighboring gene, encoding Mip, is completely retained in all vertebrates include mammals. It should be noted that the abnormal C-AQPs in vertebrates were clustered into a separated branch (Fig. 2 and Additional file 2: Figure S2a). Studies of the abnormal C-AQPs in teleost showed the permeation properties were typically elicited by AQGP [34]. Moreover, the abnormal C-AQPs in different teleost showed distinct biophysical properties. Further analysis revealed that the abnormal C-AQPs in teleost fishes play important osmoregulatory roles in piscine seawater adaptation [34]. Except that, no study has been reported for the functional of the abnormal C-AQPs in vertebrates, especially in Sauropsida. Unlike the abnormal C-AQPs in teleost that was widely expressed in different tissues or organs [34], the ortholog of AQPc2 in *Xla* genome was only trace expressed in brain, eyes and testis (FPKM < 0.5, Additional file 14: Table S3) and not expressed throughout the embryo development stages (Additional file 10: Figure S10 and Additional file 11: Figure S11). It can be inferred that the orthologs of AQPc2 may be in the transitional stage of losing during the process of evolution.

Further analysis revealed that the AQP orthologs that with one copy absent in *Xla* genome were also prone to loss during the process of subsequent evolution. As in the ortholog of AQPc2 that described above, similar phenomenon also occurs in the orthologs from AQPc3 to -c6 that were localized at the adjacent position in the second chromosomes and arise from tandem duplication in *Xenopus* species. As a comparison, the corresponding regions in higher organisms contain only two to three tandemly duplicated AQP genes [12]. Moreover, similar arrangement of C-AQPs was not detected in the genomes of fish families either. Unexpectedly, the corresponding region in reptiles was inverted in comparison to that in amphibians (Fig. 5). It should be noted that the inversion in reptiles separated the four tandemly duplicated AQP genes into two groups. Previous study indicated that the orthologs of AQP6 were lost in avian lineages during the process of evolution [12]. Synteny analyses revealed that the inversion in this region in reptile result in the deletion of AQP6 in birds (Fig. 5). Remarkably, low collinearity in this region have been detected between chicken and human. Nevertheless, synteny analyses showed extensive collinearity of this region among amphibians, platypus and human (Fig. 5). Additionally, these data confirmed that the species in Mammalia and Sauropsida have been independently evolved after the ancient amphibians. The insertion of PINLYP between the orthologs of AQPg1 and -g2 in Sauropsida also supports this conclusion (Fig. 8a). These data suggested that, in addition to duplicated by allotetraploidization, the members that arise from tandem duplication in amphibians were also prone to loss during the subsequent evolution.

Furthermore, the expansion of the orthologs from AQPc8 to -c10 also contributed to the expansion of this subfamily in amphibians (Fig. 6b). Unlike the ortholog of AQPc8, the other two orthologs only appeared in amphibians. Unexpectedly, the orthologs of AQPc9 and -c10 were also not appeared in *L. chalumnae*. Although the AQP family was highly diversified in fish families due to additional one or two rounds of WGD, the orthologs of AQPc9 and -c10 are also not detected in their genomes. As mentioned above, the orthologs from AQPc8 to -c10 in amphibians were not adjacently localized in the chromosome and not arisen from tandem duplication (Fig. 4f and Fig. 6b). High identities among these orthologs suggested that they may share the common ancestor (Additional file 5: Figure S5 and Additional file 6: Figure S6). Unfortunately, the lack of available genome sequence for the ancient amphibians prevent us to explore the origin of these orthologs. Unlike the deletion of AQPc2 and AQPc6 in higher vertebrates that they have experienced a process referred to as pseudogenization (Additional file 3: Figure S3), the orthologs of AQPc9 and -c10 have been completely reserved in *Xla* genome. Therefore, the deletion of these two orthologs in higher vertebrates warrants further research.

Similar phenomena also occurred in the AQP8 subfamily. Interestingly, this subfamily was only expanded in amphibian species, but not in the higher vertebrates. The sequences encoding the independently evolved orthologs of AQP8.1 and AQP8.2 were localized in the separated regions of the ninth pair chromosomes in *Xla* genome and in the separated chromosomes in *Xtr* genomes respectively (Fig. 4c). Synteny analysis showed that the two separated regions were extensively conserved when compared with the consistent regions in the genomes of coelacanth, the ancestor of ancient amphibians, as well as the higher vertebrates like tortoise and chicken (Fig. 7). In contrast, the sequence encoding the orthologs of AQP8.2 was absented in the conserved region in both ancient and higher vertebrates (Fig. 7b). Expression profiling revealed that AQP8.2 was not expressed in different tissue and at developmental stages (FPKM < 0.5, Additional file 14: Table S3) (Additional file 10: Figure S10 and Additional file 11: Figure S11). Combined with the pseudogenization of AQP8.2 in *Xla. L* homeolog, we could deduce that the orthologs of AQP8.2 were also at a transitional stage and prone to lose during the process of evolution. Interestingly, the ortholog of AQP8.2 was also missing in *L. chalumnae*. While the lack of available genome databases for ancient amphibian prevents us to explore the origin of AQP8.2 in amphibians.

Except the highly conserved orthologs of AQPg1 and -g2 that raised from tandem duplication and AQPg4 that localized at another chromosome (Fig. 8), synteny analysis was not performed on the remaining orthologs (AQPg3 and -g5) in AQGP subfamily due to the low collinearity between different species. While previous studies indicated that the orthologs of AQP10 have been lost or pseudogenized in turtles and some species in rodents as well as ruminants [12, 35, 36]. Similar to the orthologs that mentioned above, these pseudogenized genes may represent a transitional stage in their way to deletion.

Gene duplication, as we know, plays an important role in the evolution of genome [13, 37, 38]. Moreover, the polyploidies usually possess strong plasticity and has the capability of forming new species in plants [39, 40]. Considering the instability of the neopolyploidies, a process referred to as diploidization usually occurred during the subsequent evolution [41]. The redundant genes in polyploid are prone to revert to single copy during the process of diploidization. Polyploidization and subsequent pseudogenization is an efficient way for the deletion of abundant duplicated genes [42]. Comparative genomic study between *Xla* and *Xtr* and the other vertebrates revealed that the deletion of AQP genes in *Xla* genome laid the foundation for the AQP family status in higher vertebrates. Therefore, the polyploidization of *Xla* is an important event during the evolution process of vertebrates. In summary, gene duplication and subsequent pseudogenization and loss have played important roles in the process of AQP evolution. However, the origin and deletion as well as their functions of the orthologs from AQPc8 to -c10 in amphibians are still intriguing questions that warrant further research in the future.

## Conclusion

In summary, we analyzed the whole AQP families in an allotetraploid frog (*Xla*) and a diploid frog (*Xtr*) respectively. Phylogenetic analysis revealed the diversified AQP family in *Xenopus* species. Synteny analysis between the *Xtr* genome and the homeologs in *Xla* revealed the distribution and deletion of the AQP orthologs that arise from genome duplication. Comparative genomic research demonstrated that the duplication and subsequent deletion of AQP genes in *Xla* genome promote the evolution of the AQP family in higher vertebrates. Expression patterns of the whole AQP family in different adult tissues and throughout the embryonic development stages suggested the extensive diversification of its members in function. Briefly, this study revealed a comprehensive understanding of the AQP family in duplication and deletion during the process of evolution.

## Methods

### AQPs identification and characteristics analyses

Available genome sequences (Additional file 15: Table S4) and the annotation files from the NCBI database were employed to identify the AQP genes in *Xla* and *Xtr*. Considering the conservation of AQP genes in NPA motif, the protein sequences containing the fourteen human AQP genes were used as queries to pre-identify the putative AQP genes from the two frog genome sequences by local Tblastn program [43]. The signature of conserved NPA motifs were regarded as the characteristic for the identification of AQP genes. Meanwhile, the AQP genes of the other vertebrates (Additional file 15: Table S4) were de novo detected as described above. To ensure that all AQP repertoire were detected, the pre-identified AQP genes in all vertebrates were summarized and used as queries to re-blast the vertebrate genomes. Next, the identified AQP protein sequences were revised using NCBI protein blast online service. In addition, the AQP sequences in *Xenopus* were also confirmed using the assembly of transcriptome databases. The complete protein sequences of the identified AQPs were aligned using Clustal X2 [44]. The alignment result was visualized by DNAMAN 9 software. The amino acids residue composition of the two signatures (NPA motif and Ar/R region) in the protein sequences were summarized based on the alignment data, with the identities between different sequences were calculated simultaneously. In addition, the orthologs of the identified AQP protein sequences (except the pseudogenes in *Xla* genome) were verified by both maximum likelihood (ML) and neighbor-joining (NJ) phylogenetic analysis using MEGA 7 software [45]. Finally, the phylogenetic relationship between the amino-terminal (N-ter) half and carboxy-terminal (C-ter) half of the protein sequences was also produced using ML methods at the same time.

### Gene structure and distribution analysis

The exon/intron structures of the AQP genes in *Xla* and *Xtr* genome were analyzed by the online service named Gene Structure Display Server (GSDS 2.0) following the gene annotation and position files [46]. The chromosomal location of the identified AQP genes in different vertebrates were performed by TBtools depending on the information of gene position files [47]. The complete set of the AQP genes were renamed based on their distribution on chromosomes and the subfamilies that they were clustered.

### Duplication and synteny analysis

The synteny relationship and duplication events of the identified AQPs in the subgenomes of allotetraploid frog *Xla* (both the homeologs of *Xla. L* and *Xla.S*) and the related diploid frog *Xtr* genome were analyzed and visualized by TBtools with the default parameters. In addition, the deletion of the orthologs copies of the AQP genes in *Xla* were further analyzed based on the synteny analysis results. The pseudogenes in *Xla* genome were detected using local Blastn and Tblastn programs. The corresponding AQP protein sequences and CDS in the *Xtr* genome were used as queries. Comparative genomic studies between different species (Additional file 15: Table S4) were also performed by TBtools with the default parameters.

### Homology modeling and pore analysis

The three-dimensional structures of the identified AQPs in *Xla* were predicted using the online service named Swiss-model [48]. The structures of C-AQPs were modeled based on the crystal structure of Human AQP-4 (PDB ID: 3gd8). The structures of AQP-8 s were modeled based on the crystal structure of *Arabidopsis thaliana* ammonia permeable AQP AtTIP2;1 (PDB ID: 5i32). The structure of AQGPs were modeled based on the crystal structure of the *E.coli* glycerol facilitator (Glpf) (PDB ID: 1fx8). The inner-surface and radius of the pore in the identified AQPs were analyzed using Hole2 program [49]. The triangulated inner-surfaces were produced and visualized in VMD1.9.2 software [50].

### Expression data analysis

In order to analysis the expression pattern of the AQPs in *Xenopus* species, Illumina HiSeq SRA data containing 23 oocyte/developmental stages and 14 different adult tissues and organs of the allotetraploid frog *Xla* were download from NCBI database (Additional file 16: Table S5). These data were analyzed using Hisat2 [51] and Stringtie v1.3 [52] software as previously described. Additionally, these transcriptome data were also assembled using Stringtie software based on the genome sequence. The complete sequence of the AQP genes in *Xenopus* were confirmed by the assembly of transcriptome. The value of transcripts per million (TPM) and fragments per kilobase million (FPKM) were selected to estimate the gene expression levels of the identified AQPs respectively. The expression patterns were compared and visualized using Mev 4.9.0 software [53].

## Supplementary information

**Additional file 1: Figure S1.** Distribution and correlation of the AQP family in different vertebrates.

**Additional file 2: Figure S2.** Phylogenetic analysis of the AQP genes in vertebrates. (A) Neighbor-Joining based phylogenetic tree of the AQP genes in different vertebrates. (B) Maximum likelihood based phylogenetic tree of the amino terminal and carboxy terminal of the AQP protein sequences in different vertebrates. The AQP families were distinct separated into four clades (marked with different colors), representing the four subfamilies respectively. (C) Identities between the amino acid sequences of AQP genes in different vertebrates. (D) The conserved amino acids in the two NPA motifs. The protein sequences were collected from the following species: *Latimeria chalumnae* (*Lch*), *Rhinatrema bivittatum* (*Rbi*), *Xenopus laevis* (*Xla*), *Xenopus tropicalis* (*Xtr*), *Gopherus evgoodei* (*Gev*), *Gallus gallus* (*Gga*), *Ornithorhynchus anatinus* (*Oan*), *Homo sapiens* (*Hsa*).

**Additional file 3: Figure S3.** The retained exons and the translated amino acid sequences of the absented AQPs in *X. laevis* genome. (A) Alignment of the retained exon encoding AQPg3 and translated amino acid sequence in *Xla. S* homeolog. (B) Alignment of the retained exons encoding AQPc6 and translated amino acid sequence in *Xla. L* homeolog. (C) Alignment of the retained exons encoding AQPg5 and translated amino acid sequence in *Xla. S* homeolog. (D) Alignment of the retained exons encoding AQP8.2 and translated amino acid sequence in *Xla. L* homeolog. In this figure, in-frame stop codons were marked with red “*”. Single nucleotide insertions were marked with red arrow. Single nucleotide deletion was marked with red line.

**Additional file 4: Figure S4.** Synteny analyses of the corresponding region in the genome of *Xla* and *Xtr*. (A) Collinearity analysis of the region in the end terminal of the second chromosome in *Xla* and some scaffolds in *Xtr*. (B) Collinearity analysis of the region in the end terminal of the eighth chromosome in *Xla* and some scaffolds in *Xtr*. (C) Collinearity analysis between the ninth pair chromosomes in *Xla* genome. Part A represent the region corresponding to the tenth chromosome in *Xtr* genome. Part B represent the region corresponding with the ninth chromosome in *Xtr* genome. Fusion region represent the genes in this region were not detected in both the ninth and tenth chromosomes in *Xtr* genome. (D) Collinearity analysis of the fusion region in *Xla* genome and some scaffolds in *Xtr* genome.

**Additional file 5: Figure S5.** Identities between the CDS encoding AQP genes in the homeologs of *X. laevis* and *X. tropicalis* genome. (A) Heatmap of the identities between the sequence of AQP CDS in *Xla* and *Xtr* genome. (B) Identity between the ortholog AQP genes in *Xla* and *Xtr* genome.

**Additional file 6: Figure S6.** Identities between the amino acid sequences of AQP genes in the homeologs of *X. laevis* and *X. tropicalis* genome. (A) Heatmap of the identities between the amino acid sequence of AQP in *Xla* and *Xtr* genome. (B) Identity of the amino acid sequences between the ortholog AQPs in *Xla* and *Xtr* genome.

**Additional file 7: Figure S7.** (A) Multiple sequence alignment of CDS encoding AQPs in *Xla* genome. The nucleotides highlighted in pink are conserved more than 75%. The nucleotides highlighted in cyan are conserved more than 50%. (B) The conserved nucleotide sequence encoding the first NPA motif. (C) The conserved nucleotide sequence encoding the second NPA motif.

**Additional file 8: Figure S8.** Multiple sequence alignment of amino acid sequences of the AQP genes in *X. laevis* genome. Different subfamilies were separated by different colored boxes. The amino acids highlighted in black are completely conserved in all sequences. The amino acids highlighted in pink are conserved more than 75%. The amino acids highlighted in cyan are conserved more than 50%. The transmembrane regions were marked with purple helixes. The re-entrant regions were marked with green helixes. The two conserved NPA motifs were boxed in red. The amino acids that constituted the Ar/R region were boxed in black and marked with red stars.

**Additional file 9: Figure S9.** Pore pattern analyses of the AQPs in *X. laevis* genome. (A) Inner surface of the AQP proteins that clustered into C-AQP subfamily in *Xla* genome. (B) Individual diameter profiles of the AQP protein channel that clustered into C-AQP subfamily. (C) Comparison of the structure of the Ar/R region between AQPc1 and AQPc2. (D) Inner surface of the AQP proteins that clustered into AQGP subfamily in *Xla* genome. (E) Individual diameter profiles of the AQP protein channel that clustered into AQGP subfamily. (F) Inner surface of the AQP proteins that clustered into AQP-8 subfamily in *Xla* genome. (G) Individual diameter profiles of the AQP protein channel that clustered into AQP-8 subfamily.

**Additional file 10: Figure S10.** Heatmap of the AQP family expression pattern throughout the embryo development stages of *X. laevis*.

**Additional file 11: Figure S11.** Heatmap of the AQP family expression pattern in different tissues or organs of adult *X. laevis*.

**Additional file 12: Table S1.** Summary of the AQP families in different vertebrates.

**Additional file 13: Table S2.** Basic characteristic of the AQP families in the genome of *X. laevis* and *X. tropicalis*.

**Additional file 14: Table S3.** Expression of AQP family in different organs and at different development stages in *X. laevis* genome.

**Additional file 15: Table S4.** Summary of the genome sequence data used in this study.

**Additional file 16: Table S5.** Summary of the RNAseq data used in this study.

## Data Availability

The genome data and annotation files of the different vertebrates (Additional file 15: Table S4) were downloaded from NCBI. The Illumina HiSeq SRA data containing 23 oocyte/developmental stages and 14 different adult tissues or organs (Additional file 16: Table S5) of the allotetraploid frog *X. laevis* were downloaded from NCBI. All data generated or analyzed during this study are included in this published article and its supplementary information files.

## Abbreviations

AQP: Aquaporin
*Xla*: *Xenopus laevis*
*Xtr*: *Xenopus tropicalis*
*Xla.L*: The longer homeolog in *Xla* genome
*Xla.S*: The shorter homeolog in *Xla* genome
TPM: Transcripts per million
FPKM: Fragments per kilobase million
