# Supplementary figures and images for: Polyploidization and pseudogenization in allotetraploid frog Xenopus laevis promote the evolution of aquaporin family in higher vertebrates
Source: BMC Genomics. 2020 Jul 29;21:525. doi: 10.1186/s12864-020-06942-y (PMC7392679; doi:10.1186/s12864-020-06942-y)

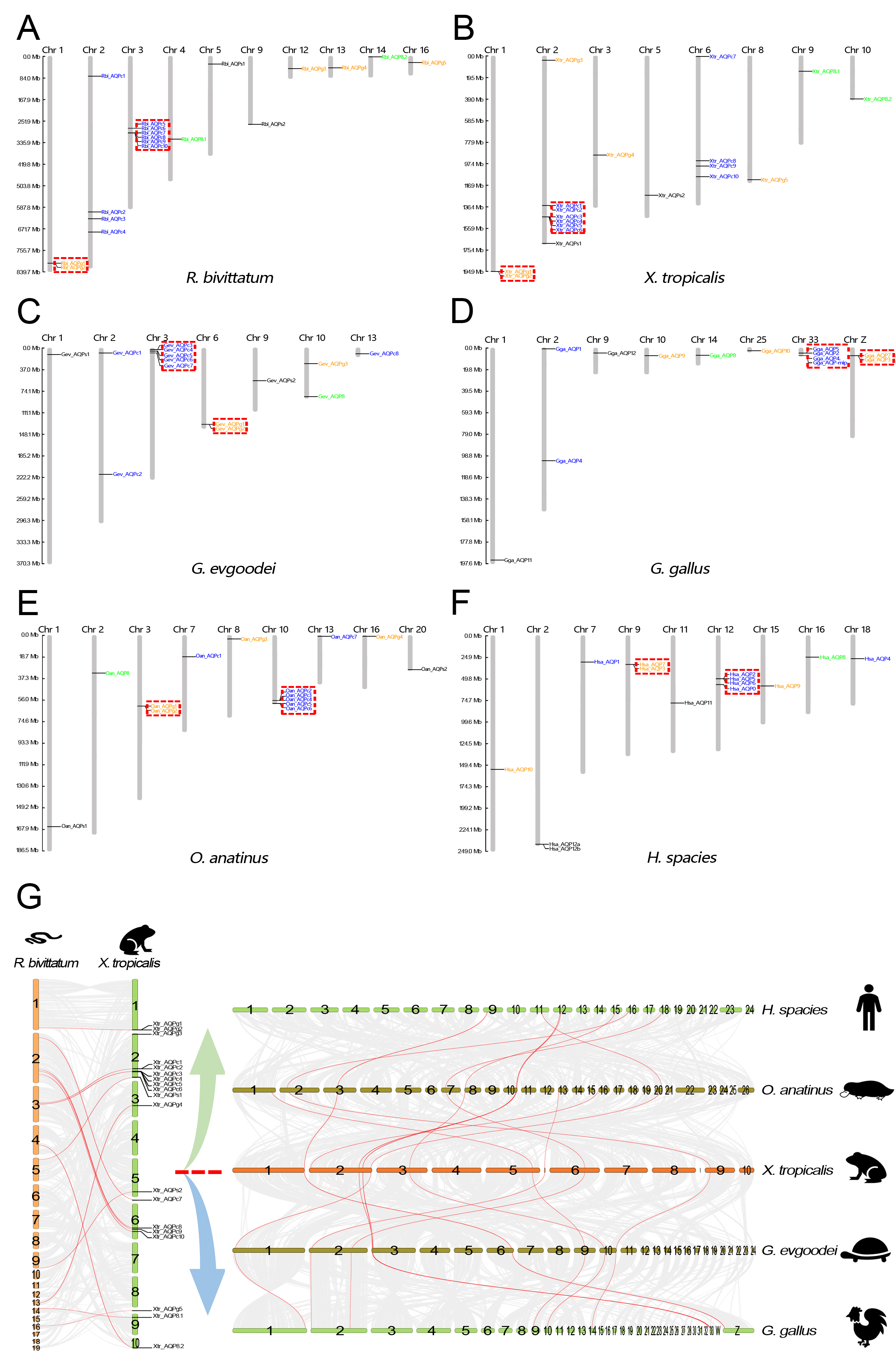

Supplement: Supplementary file 1 — Additional file 1: Figure S1. Distribution and correlation of the AQP family in different vertebrates. [file 12864_2020_6942_MOESM1_ESM.tif]

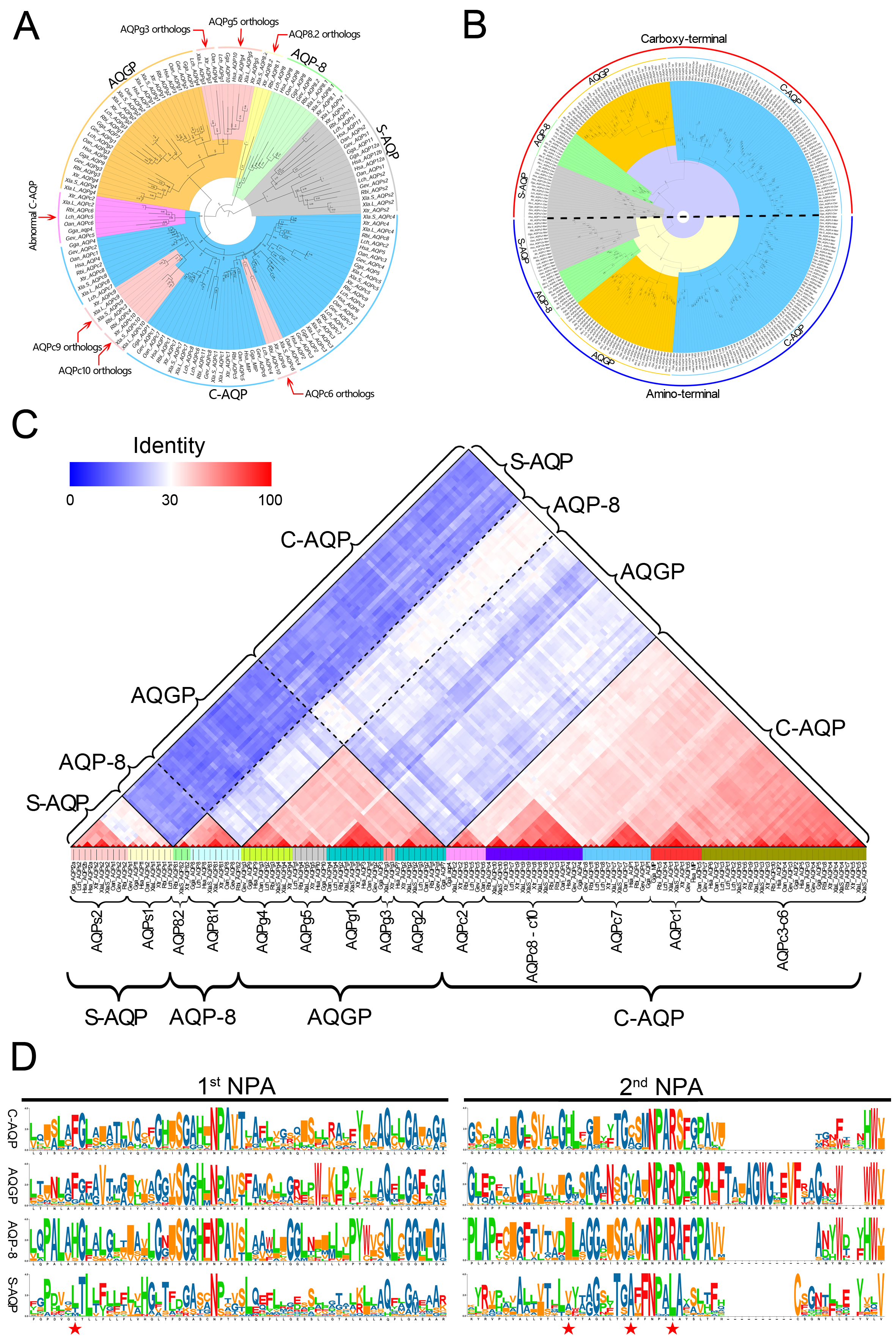

Supplement: Supplementary file 2 — Additional file 2: Figure S2. Phylogenetic analysis of the AQP genes in vertebrates. (A) Neighbor-Joining based phylogenetic tree of the AQP genes in different vertebrates. (B) Maximum likelihood based phylogenetic tree of the amino terminal and carboxy terminal of the AQP protein sequences in different vertebrates. The AQP families were distinct separated into four clades (marked with different colors), representing the four subfamilies respectively. (C) Identities between the amino acid sequences of AQP genes in different vertebrates. (D) The conserved amino acids in the two NPA motifs. The protein sequences were collected from the following species: Latimeria chalumnae (Lch), Rhinatrema bivittatum (Rbi), Xenopus laevis (Xla), Xenopus tropicalis (Xtr), Gopherus evgoodei (Gev), Gallus gallus (Gga), Ornithorhynchus anatinus (Oan), Homo sapiens (Hsa). [file 12864_2020_6942_MOESM2_ESM.tif]

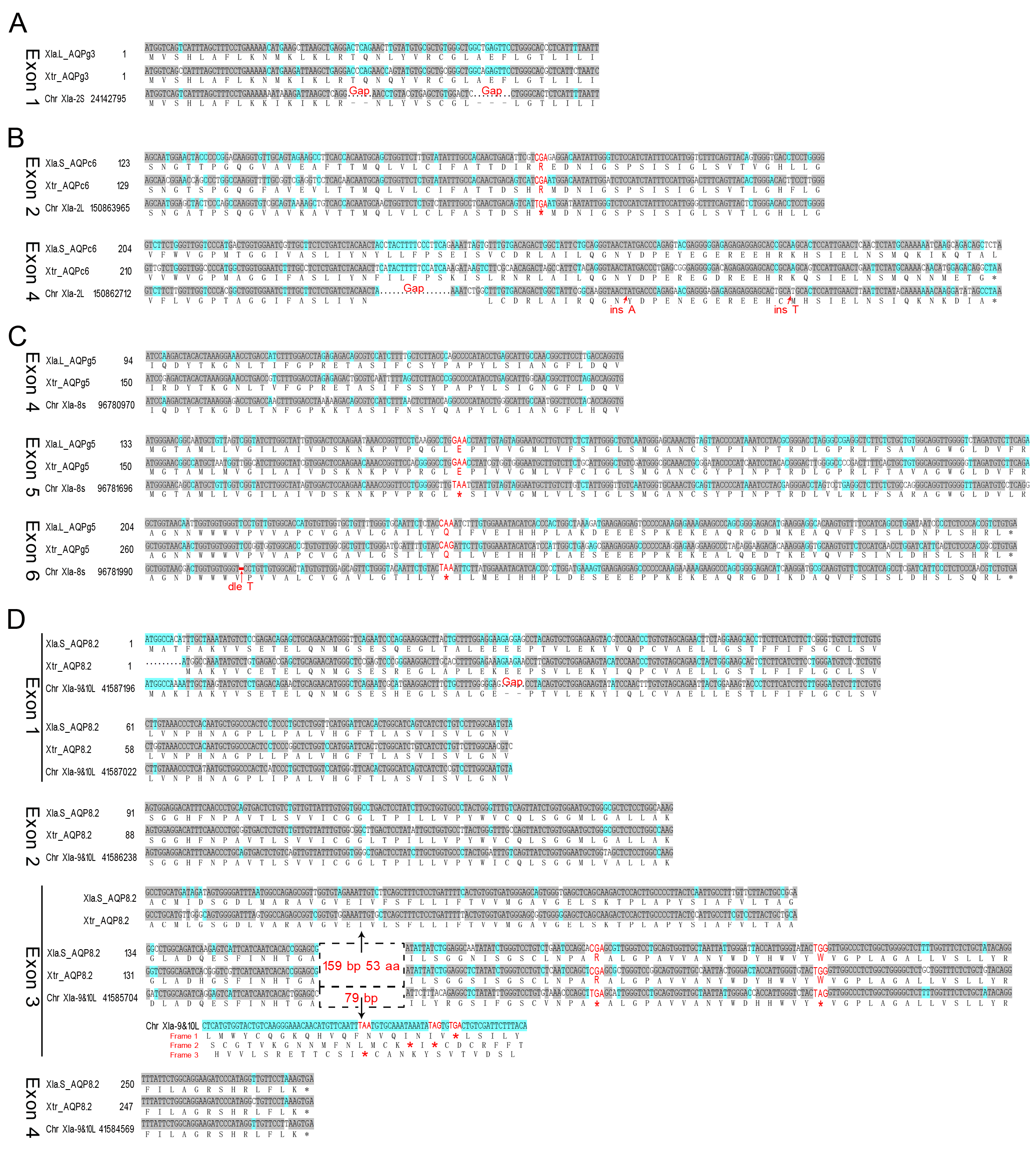

Supplement: Supplementary file 3 — Additional file 3: Figure S3. The retained exons and the translated amino acid sequences of the absented AQPs in X. laevis genome. (A) Alignment of the retained exon encoding AQPg3 and translated amino acid sequence in Xla. S homeolog. (B) Alignment of the retained exons encoding AQPc6 and translated amino acid sequence in Xla. L homeolog. (C) Alignment of the retained exons encoding AQPg5 and translated amino acid sequence in Xla. S homeolog. (D) Alignment of the retained exons encoding AQP8.2 and translated amino acid sequence in Xla. L homeolog. In this figure, in-frame stop codons were marked with red “*”. Single nucleotide insertions were marked with red arrow. Single nucleotide deletion was marked with red line. [file 12864_2020_6942_MOESM3_ESM.tif]

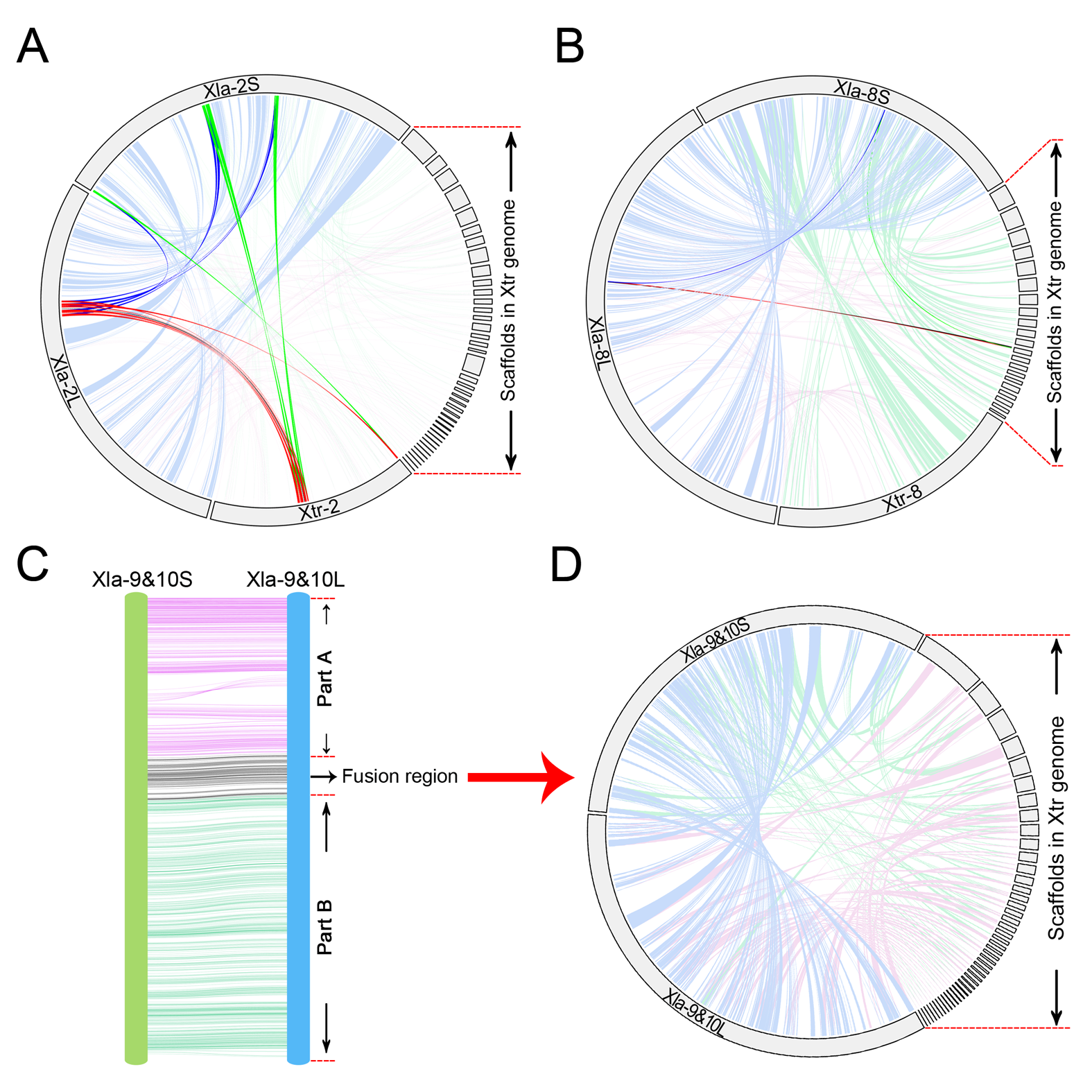

Supplement: Supplementary file 4 — Additional file 4: Figure S4. Synteny analyses of the corresponding region in the genome of Xla and Xtr. (A) Collinearity analysis of the region in the end terminal of the second chromosome in Xla and some scaffolds in Xtr. (B) Collinearity analysis of the region in the end terminal of the eighth chromosome in Xla and some scaffolds in Xtr. (C) Collinearity analysis between the ninth pair chromosomes in Xla genome. Part A represent the region corresponding to the tenth chromosome in Xtr genome. Part B represent the region corresponding with the ninth chromosome in Xtr genome. Fusion region represent the genes in this region were not detected in both the ninth and tenth chromosomes in Xtr genome. (D) Collinearity analysis of the fusion region in Xla genome and some scaffolds in Xtr genome. [file 12864_2020_6942_MOESM4_ESM.tif]

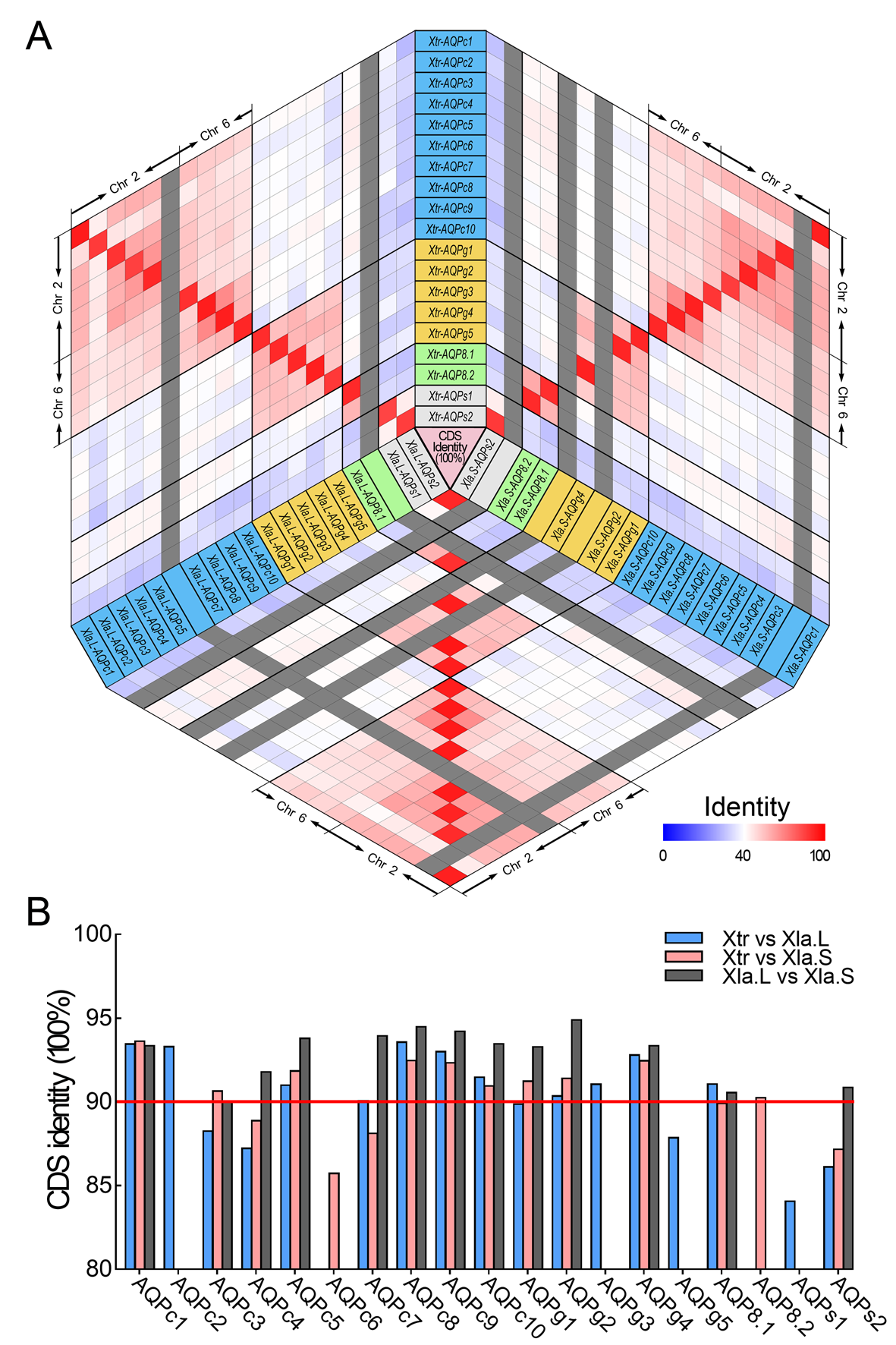

Supplement: Supplementary file 5 — Additional file 5: Figure S5. Identities between the CDS encoding AQP genes in the homeologs of X. laevis and X. tropicalis genome. (A) Heatmap of the identities between the sequence of AQP CDS in Xla and Xtr genome. (B) Identity between the ortholog AQP genes in Xla and Xtr genome. [file 12864_2020_6942_MOESM5_ESM.tif]

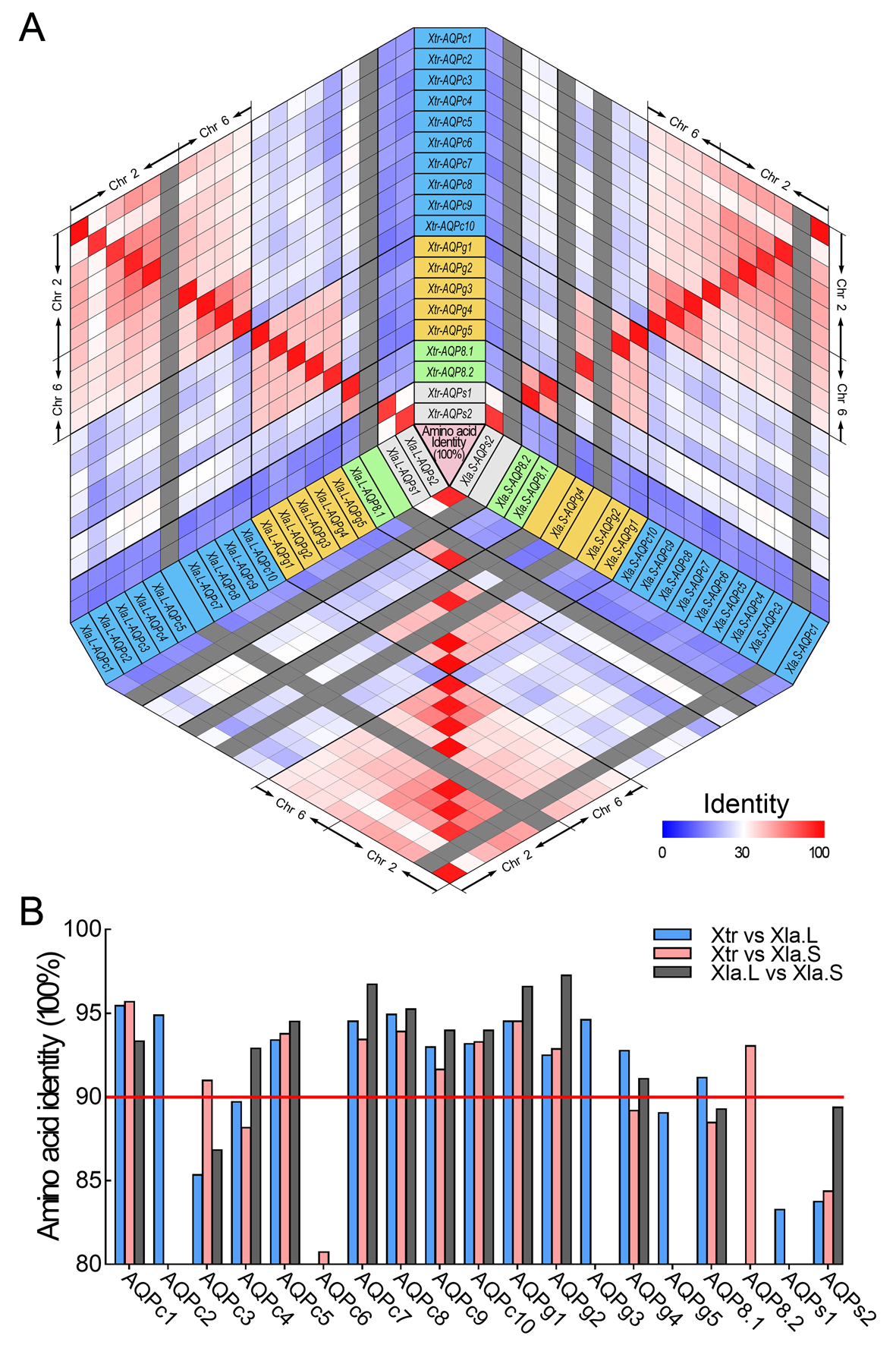

Supplement: Supplementary file 6 — Additional file 6: Figure S6. Identities between the amino acid sequences of AQP genes in the homeologs of X. laevis and X. tropicalis genome. (A) Heatmap of the identities between the amino acid sequence of AQP in Xla and Xtr genome. (B) Identity of the amino acid sequences between the ortholog AQPs in Xla and Xtr genome. [file 12864_2020_6942_MOESM6_ESM.tif]

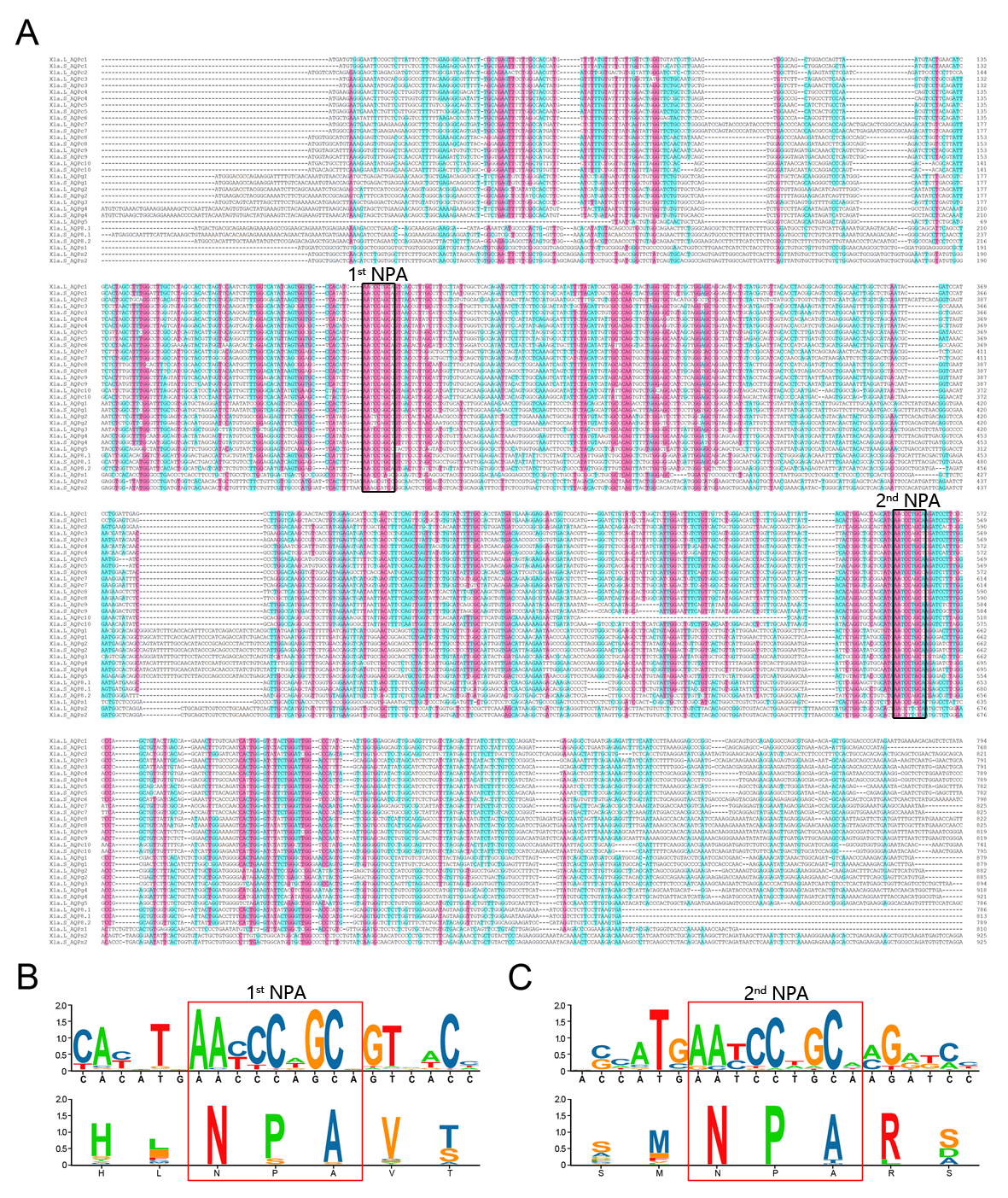

Supplement: Supplementary file 7 — Additional file 7: Figure S7. (A) Multiple sequence alignment of CDS encoding AQPs in Xla genome. The nucleotides highlighted in pink are conserved more than 75%. The nucleotides highlighted in cyan are conserved more than 50%. (B) The conserved nucleotide sequence encoding the first NPA motif. (C) The conserved nucleotide sequence encoding the second NPA motif. [file 12864_2020_6942_MOESM7_ESM.tif]

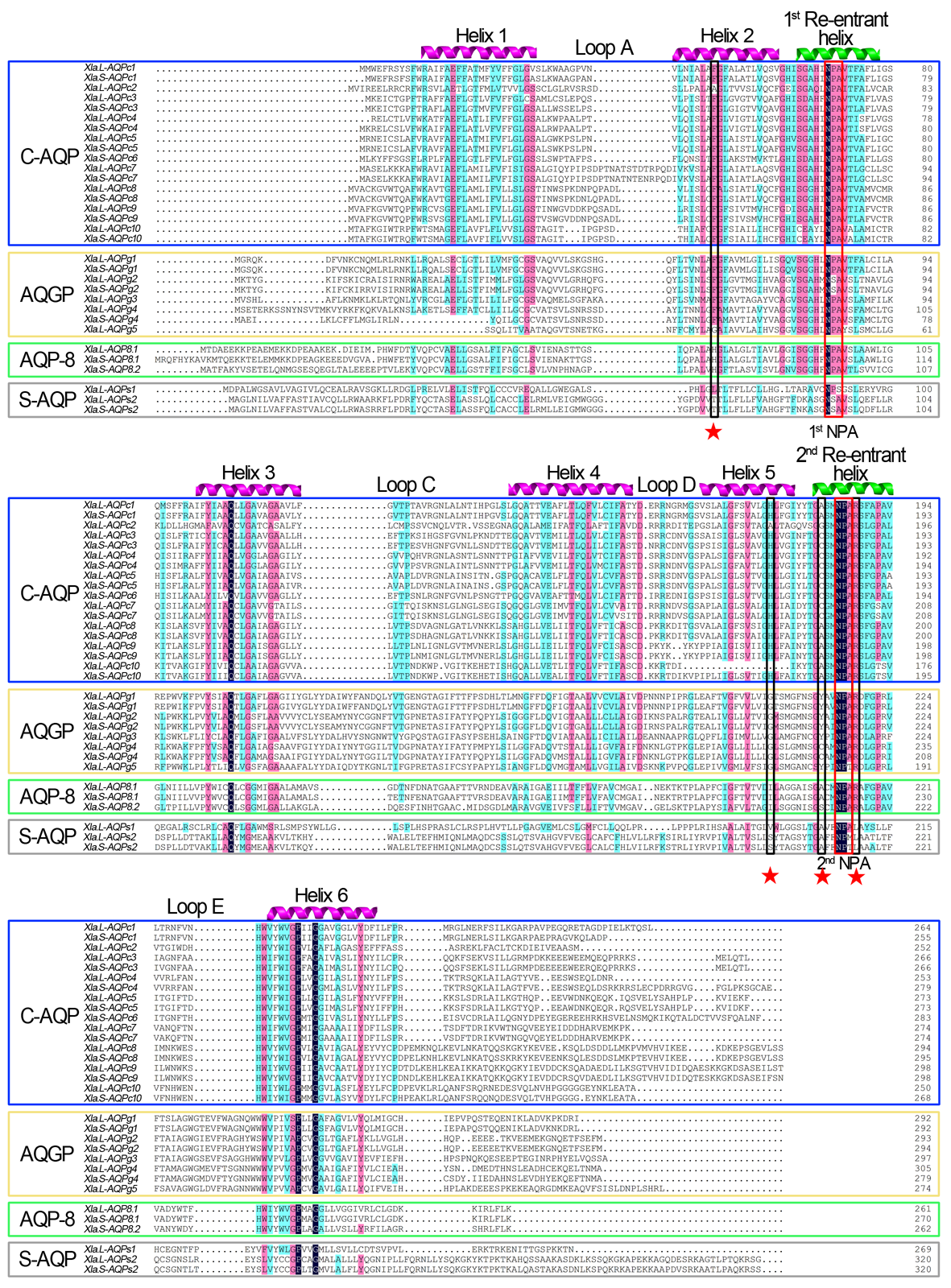

Supplement: Supplementary file 8 — Additional file 8: Figure S8. Multiple sequence alignment of amino acid sequences of the AQP genes in X. laevis genome. Different subfamilies were separated by different colored boxes. The amino acids highlighted in black are completely conserved in all sequences. The amino acids highlighted in pink are conserved more than 75%. The amino acids highlighted in cyan are conserved more than 50%. The transmembrane regions were marked with purple helixes. The re-entrant regions were marked with green helixes. The two conserved NPA motifs were boxed in red. The amino acids that constituted the Ar/R region were boxed in black and marked with red stars. [file 12864_2020_6942_MOESM8_ESM.tif]

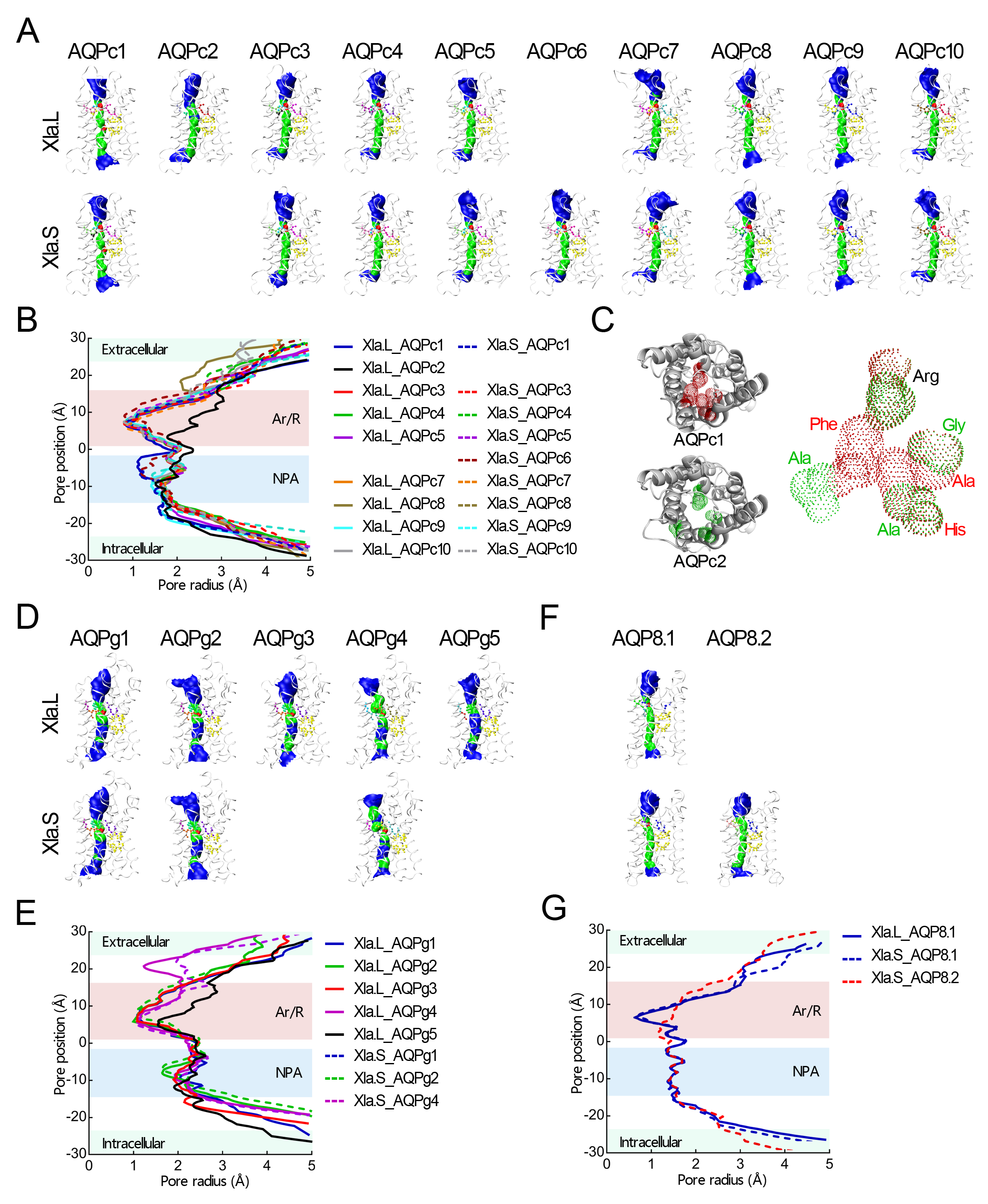

Supplement: Supplementary file 9 — Additional file 9: Figure S9. Pore pattern analyses of the AQPs in X. laevis genome. (A) Inner surface of the AQP proteins that clustered into C-AQP subfamily in Xla genome. (B) Individual diameter profiles of the AQP protein channel that clustered into C-AQP subfamily. (C) Comparison of the structure of the Ar/R region between AQPc1 and AQPc2. (D) Inner surface of the AQP proteins that clustered into AQGP subfamily in Xla genome. (E) Individual diameter profiles of the AQP protein channel that clustered into AQGP subfamily. (F) Inner surface of the AQP proteins that clustered into AQP-8 subfamily in Xla genome. (G) Individual diameter profiles of the AQP protein channel that clustered into AQP-8 subfamily. [file 12864_2020_6942_MOESM9_ESM.tif]

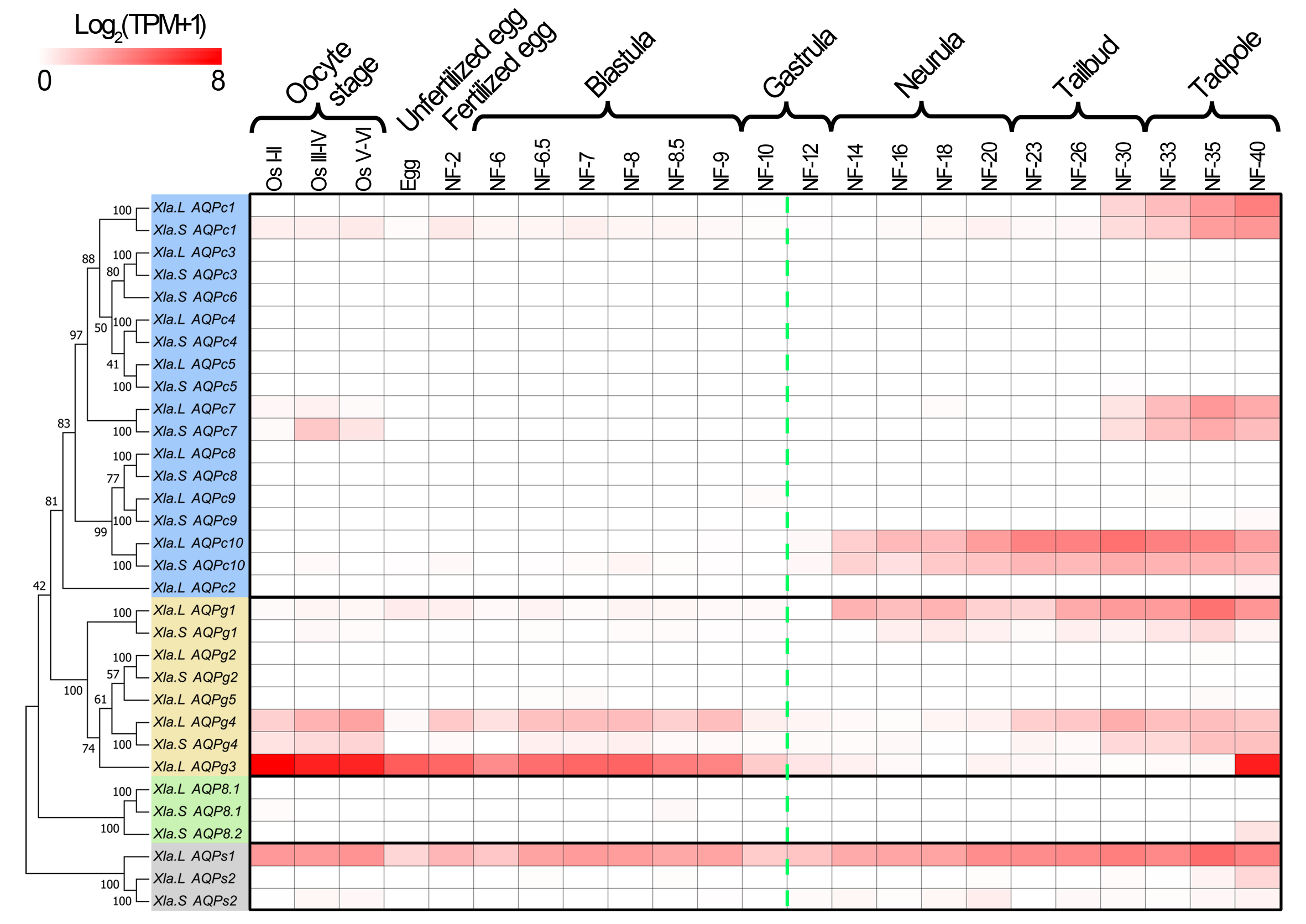

Supplement: Supplementary file 10 — Additional file 10: Figure S10. Heatmap of the AQP family expression pattern throughout the embryo development stages of X. laevis. [file 12864_2020_6942_MOESM10_ESM.tif]

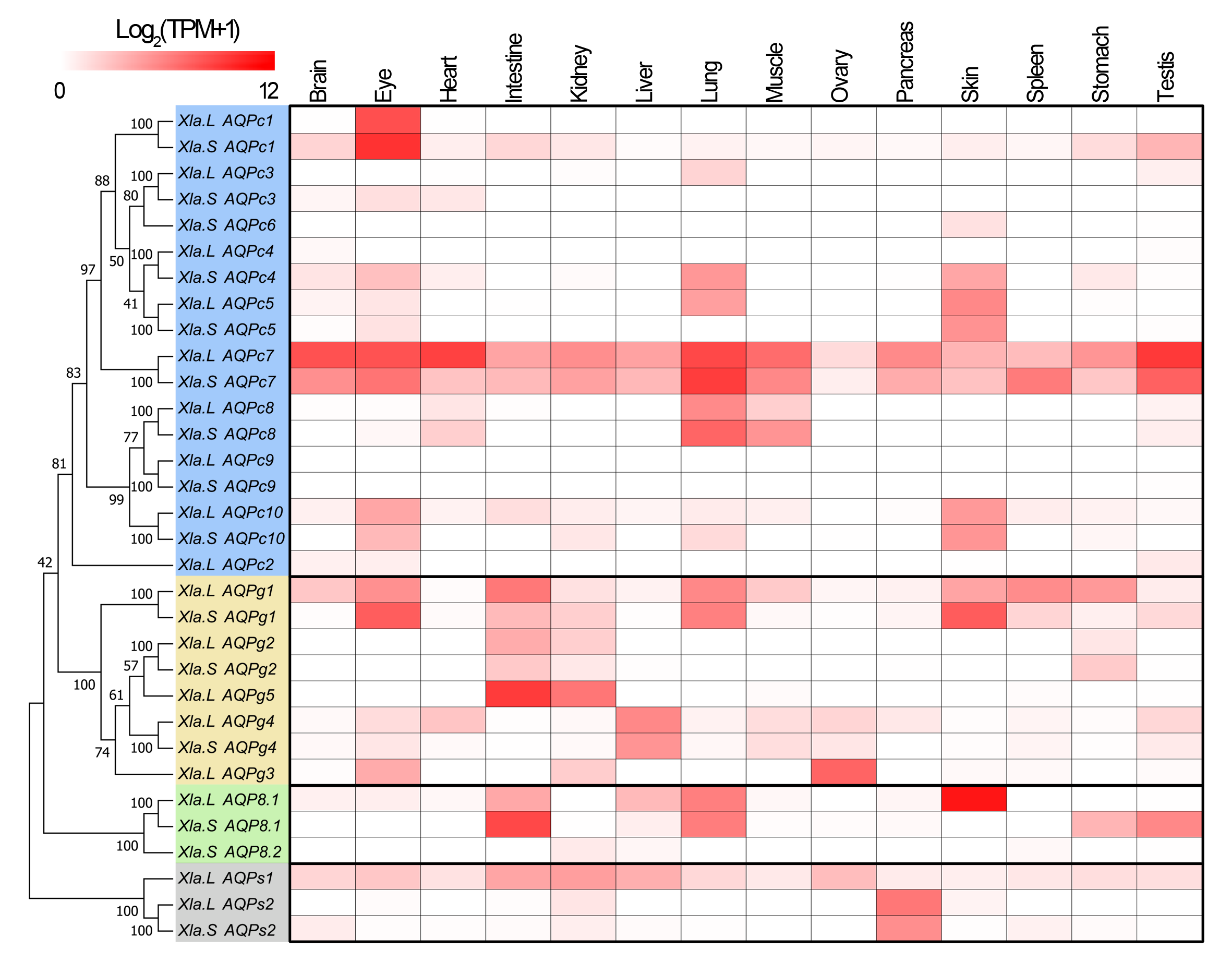

Supplement: Supplementary file 11 — Additional file 11: Figure S11. Heatmap of the AQP family expression pattern in different tissues or organs of adult X. laevis. [file 12864_2020_6942_MOESM11_ESM.tif]
