# Supplementary material for: Polyploidization and pseudogenization in allotetraploid frog Xenopus laevis promote the evolution of aquaporin family in higher vertebrates
Source: BMC Genomics. 2020 Jul 29;21:525. doi: 10.1186/s12864-020-06942-y (PMC7392679; doi:10.1186/s12864-020-06942-y)
Supplement: Supplementary file 12 — Additional file 12: Table S1. Summary of the AQP families in different vertebrates. [file 12864_2020_6942_MOESM12_ESM.doc]

**S1 Table. Summary of the AQP families in the genome of the other vertebrates.**

|  | ***R. bivittatum*** | ***X. tropicalis*** | ***X. laevis*** | | ***G. evgoodei*** | ***G. gallus*** | ***O. anatinus*** | ***H. sapiens*** |
| --- | --- | --- | --- | --- | --- | --- | --- | --- |
|  |  |  | ***Xla.L*** | ***Xla.S*** |  |  |  |  |
| C-AQP | 11 | 10 | 9 | 9 | 8 | 6 | 7 | 6 |
| AQGP | 4 | 5 | 5 | 3 | 3 | 4 | 4 | 4 |
| AQP-8 | 2 | 2 | 1 | 2 | 1 | 1 | 1 | 1 |
| S-AQP | 2 | 2 | 2 | 1 | 2 | 2 | 2 | 3 |
| Total | 19 | 19 | 17 | 15 | 14 | 13 | 14 | 14 |
