# Supplementary material for: Polyploidization and pseudogenization in allotetraploid frog Xenopus laevis promote the evolution of aquaporin family in higher vertebrates
Source: BMC Genomics. 2020 Jul 29;21:525. doi: 10.1186/s12864-020-06942-y (PMC7392679; doi:10.1186/s12864-020-06942-y)
Supplement: Supplementary file 13 — Additional file 13: Table S2. Basic characteristic of the AQP families in the genome of X. laevis and X. tropicalis. [file 12864_2020_6942_MOESM13_ESM.doc]

**S2 Table. Basic characteristic of the AQP families in the genome of *Xenopus laevis* and *Xenopus tropicalis*.**

| **Subfamily** | **Accession number** | **Gene names** | **Chromosome** | **1^st^ NPA** | **2^nd^ NPA** | **Ar/R region** | | | |
| --- | --- | --- | --- | --- | --- | --- | --- | --- | --- |
|  |  |  |  |  |  | **He_2_** | **He_5_** | **Le_1_** | **Le_2_** |
| C-AQP | NP_001090816.1 | Xtr_AQPc1 | 2 | NPA | NPA | F | H | A | R |
|  | NP_001088304.1 | Xla.L_AQPc1 | 2L | NPA | NPA | F | H | A | R |
|  | NP_001089398.1 | Xla.S_AQPc1 | 2S | NPA | NPA | F | H | A | R |
|  | XP_012813766.1 | Xtr_AQPc2 | 2 | NPA | NPA | A | A | G | R |
|  | XP_018101033.1 | Xla.L_AQPc2 | 2L | NPA | NPA | A | A | G | R |
|  | XP_002935778.1 | Xtr_AQPc3 | 2 | NPA | NPA | F | H | C | R |
|  | XP_018101220.1 | Xla.L_AQPc3 | 2L | NPA | NPA | F | H | C | R |
|  | NP_001137369.1 | Xla.S_AQPc3 | 2S | NPA | NPA | F | H | C | R |
|  | NP_001297041.1 | Xtr_AQPc4 | 2 | NPA | NPA | F | H | C | R |
|  | OCT95941.1 | Xla.L_AQPc4 | 2L | NPA | NPA | F | H | C | R |
|  | XP_018104235.1 | Xla.S_AQPc4 | 2S | NPA | NPA | F | H | C | R |
|  | NP_001015749.1 | Xtr_AQPc5 | 2 | NPA | NPA | F | H | C | R |
|  | XP_018101221.1 | Xla.L_AQPc5 | 2L | NPA | NPA | F | H | C | R |
|  | NP_001079331.1 | Xla.S_AQPc5 | 2S | NPA | NPA | F | H | C | R |
|  | XP_012813770.2 | Xtr_AQPc6 | 2 | NPA | NPA | F | H | C | R |
|  | NP_001163923.1 | Xla.S_AQPc6 | 2S | NPA | NPA | F | H | C | R |
|  | NP_001005829.1 | Xtr_AQPc7 | 6 | NPA | NPA | F | H | C | R |
|  | NP_001085391.1 | Xla.L_AQPc7 | 6L | NPA | NPA | F | H | C | R |
|  | NP_001088210.1 | Xla.S_AQPc7 | 6S | NPA | NPA | F | H | C | R |
|  | NP_001304774.1 | Xtr_AQPc8 | 6 | NPA | NPA | F | H | A | R |
|  | NP_001124421.1 | Xla.L_AQPc8 | 6L | NPA | NPA | F | H | A | R |
|  | XP_018079167.1 | Xla.S_AQPc8 | 6S | NPA | NPA | F | H | A | R |
|  | XP_017950427.1 | Xtr_AQPc9 | 6 | NPA | NPA | F | H | A | R |
|  | XP_018122748.1 | Xla.L_AQPc9 | 6L | NPA | NPA | F | H | A | R |
|  | XP_018079232.1 | Xla.S_AQPc9 | 6S | NPA | NPA | F | H | A | R |
|  | NP_001135583.1 | Xtr_AQPc10 | 6 | NPA | NPA | F | H | A | R |
|  | XP_018122408.1 | Xla.L_AQPc10 | 6L | NPA | NPA | F | H | A | R |
|  | XP_018079315.1 | Xla.S_AQPc10 | 6S | NPA | NPA | F | H | A | R |
| AQGP | NP_001016845.1 | Xtr_AQPg1 | 1 | NPA | NPA | F | G | Y | R |
|  | NP_001087946.1 | Xla.L_AQPg1 | 1L | NPA | NPA | F | G | Y | R |
|  | XP_018100113.1 | Xla.S_AQPg1 | 1S | NPA | NPA | F | G | Y | R |
|  | NP_001015726.1 | Xtr_AQPg2 | 1 | NSA | NPA | F | G | Y | R |
|  | XP_018082419.1 | Xla.L_AQPg2 | 1L | NSA | NPA | F | G | Y | R |
|  | XP_018100112.1 | Xla.S_AQPg2 | 1S | NSA | NPA | F | G | Y | R |
|  | XP_002940511.2 | Xtr_AQPg3 | 2 | NPA | NPA | F | G | Y | R |
|  | NP_001082310.1 | Xla.L_AQPg3 | 2L | NPA | NPA | F | G | Y | R |
|  | XP_002937719.1 | Xtr_AQPg4 | 3 | NPA | NPA | F | G | C | R |
|  | XP_018108516.1 | Xla.L_AQPg4 | 3L | NPA | NPA | F | G | C | R |
|  | XP_018110967.1 | Xla.S_AQPg4 | 3S | NPA | NPA | F | G | C | R |
|  | XP_002943404.2 | Xtr_AQPg5 | 8 | NPA | NPT | G | G | Y | R |
|  | XP_018086514.1 | Xla.L_AQPg5 | 8L | NPA | NPT | G | G | Y | R |
| AQP-8 | NP_001107728.1 | Xtr_AQP8.1 | 9 | NPA | NPA | H | I | A | R |
|  | XP_018092551.1 | Xla.L_AQP8.1 | 9/10L | NPA | NPA | H | I | A | R |
|  | XP_018094770.1 | Xla.S_AQP8.1 | 9/10S | NPA | NPA | H | I | A | R |
|  | XP_002937066.2 | Xtr_AQP8.2 | 10 | NPA | NPA | H | I | S | R |
|  | NP_001089643.1 | Xla.S_AQP8.2 | 9/10S | NPA | NPA | H | I | S | R |
| S-AQP | XP_004912256.1 | Xtr_AQPs1 | 2 | NPS | NPA | L | V | A | L |
|  | XP_018103356.1 | Xla.L_AQPs1 | 2L | NPS | NPA | L | V | A | L |
|  | XP_002935146.2 | Xtr_AQPs2 | 5 | NSA | NPT | T | S | A | R |
|  | XP_018119445.1 | Xla.L_AQPs2 | 5L | NSA | NPM | T | S | A | R |
|  | NP_001088119.1 | Xla.S_AQPs2 | 5S | NSA | NPT | T | S | A | R |
