# Supplementary material for: Polyploidization and pseudogenization in allotetraploid frog Xenopus laevis promote the evolution of aquaporin family in higher vertebrates
Source: BMC Genomics. 2020 Jul 29;21:525. doi: 10.1186/s12864-020-06942-y (PMC7392679; doi:10.1186/s12864-020-06942-y)
Supplement: Supplementary file 15 — Additional file 15: Table S4. Summary of the genome sequence data used in this study. [file 12864_2020_6942_MOESM15_ESM.doc]

**S4 Table. Summary of the genome sequence data used in this study.**

| **Organism** | **Accession** | **Assembly** | **Level** |
| --- | --- | --- | --- |
| *Latimeria chalumnae* | PRJNA56111 | GCA_000225785.1 | Scaffold |
| *Rhinatrema bivittatum* | PRJEB32111 | GCF_901001135.1 | Chromosome |
| *Xenopus laevis* | PRJNA338693 | GCA_001663975.1 | Chromosome |
| *Xenopus tropicalis* | PRJNA205740 | GCA_000004195.4 | Chromosome |
| *Gopherus evgoodei* | PRJNA489103 | GCF_007399415.2 | Chromosome |
| *Gallus gallus* | PRJNA13342 | GCF_000002315.6 | Chromosome |
| *Ornithorhynchus anatinus* | PRJNA534073 | GCA_004115215.2 | Chromosome |
| *Homo sapiens* | PRJNA31257 | GCF_000001405.39 | Chromosome |
