# Supplementary material for: Polyploidization and pseudogenization in allotetraploid frog Xenopus laevis promote the evolution of aquaporin family in higher vertebrates
Source: BMC Genomics. 2020 Jul 29;21:525. doi: 10.1186/s12864-020-06942-y (PMC7392679; doi:10.1186/s12864-020-06942-y)
Supplement: Supplementary file 16 — Additional file 16: Table S5. Summary of the RNAseq data used in this study. [file 12864_2020_6942_MOESM16_ESM.doc]

**S5 Table. Summary of the RNAseq data used in this study.**

| **Runs** | **Experiment** | **GEO_Accession** | **Description** |
| --- | --- | --- | --- |
| SRR2515135 | SRX1286458 | GSM1893239 | brain |
| SRR2515136 | SRX1286459 | GSM1893240 | eye |
| SRR2515137 | SRX1286460 | GSM1893241 | heart |
| SRR2515138 | SRX1286461 | GSM1893242 | intestine |
| SRR2515139 | SRX1286462 | GSM1893243 | kidney |
| SRR2515140 | SRX1286463 | GSM1893244 | liver |
| SRR2515141 | SRX1286464 | GSM1893245 | lung |
| SRR2515142 | SRX1286465 | GSM1893246 | muscle |
| SRR2515143 | SRX1286466 | GSM1893247 | ovary |
| SRR2515144 | SRX1286467 | GSM1893248 | pancreas |
| SRR2515145 | SRX1286468 | GSM1893249 | skin |
| SRR2515146 | SRX1286469 | GSM1893250 | spleen |
| SRR2515147 | SRX1286470 | GSM1893251 | stomach |
| SRR2515148 | SRX1286471 | GSM1893252 | testis |
| SRR2515149 | SRX1286472 | GSM1893253 | brain |
| SRR2515150 | SRX1286473 | GSM1893254 | eye |
| SRR2515151 | SRX1286474 | GSM1893255 | heart |
| SRR2515152 | SRX1286475 | GSM1893256 | intestine |
| SRR2515153 | SRX1286476 | GSM1893257 | kidney |
| SRR2515154 | SRX1286477 | GSM1893258 | liver |
| SRR2515155 | SRX1286478 | GSM1893259 | lung |
| SRR2515156 | SRX1286479 | GSM1893260 | muscle |
| SRR2515157 | SRX1286480 | GSM1893261 | ovary |
| SRR2515158 | SRX1286481 | GSM1893262 | pancreas |
| SRR2515159 | SRX1286482 | GSM1893263 | skin |
| SRR2515160 | SRX1286483 | GSM1893264 | spleen |
| SRR2515161 | SRX1286484 | GSM1893265 | stomach |
| SRR2515162 | SRX1286485 | GSM1893266 | testis |
| SRR2517984 | SRX1287719 | GSM1893595 | oocyte stage 1-2 |
| SRR2517985 | SRX1287720 | GSM1893596 | oocyte stage 3-4 |
| SRR2517986 | SRX1287721 | GSM1893597 | oocyte stage 5-6 |
| SRR2589787 | SRX1319030 | GSM1904663 | NF stage 0 |
| SRR2589788 | SRX1319031 | GSM1904664 | NF stage 2 |
| SRR2589789 | SRX1319032 | GSM1904665 | NF stage 6 |
| SRR2589790 | SRX1319033 | GSM1904666 | NF stage 6.5 |
| SRR2589791 | SRX1319034 | GSM1904667 | NF stage 7 |
| SRR2589792 | SRX1319035 | GSM1904668 | NF stage 8 |
| SRR2589793 | SRX1319036 | GSM1904669 | NF stage 8.5 |
| SRR2589794 | SRX1319037 | GSM1904670 | NF stage 9 |
| SRR2589795 | SRX1319038 | GSM1904671 | NF stage 10 |
| SRR2589796 | SRX1319039 | GSM1904672 | NF stage 12 |
| SRR2589797 | SRX1319040 | GSM1904673 | NF stage 14 |
| SRR2589798 | SRX1319041 | GSM1904674 | NF stage 16 |
| SRR2589799 | SRX1319042 | GSM1904675 | NF stage 18 |
| SRR2589800 | SRX1319043 | GSM1904676 | NF stage 20 |
| SRR2589801 | SRX1319044 | GSM1904677 | NF stage 23 |
| SRR2589802 | SRX1319045 | GSM1904678 | NF stage 26 |
| SRR2589803 | SRX1319046 | GSM1904679 | NF stage 30 |
| SRR2589804 | SRX1319047 | GSM1904680 | NF stage 33 |
| SRR2517981 | SRX1287716 | GSM1893592 | NF stage 35 |
| SRR2517999 | SRX1287734 | GSM1893610 | NF stage 35 |
| SRR2517982 | SRX1287717 | GSM1893593 | NF stage 40 |
| SRR2517996 | SRX1287731 | GSM1893607 | NF stage 40 |
